# Protocol for life cycle assessment modeling of US fruit and vegetable supply chains- cases of processed potato and tomato products

**DOI:** 10.1016/j.dib.2020.106639

**Published:** 2020-12-10

**Authors:** Ranjan Parajuli, Dave Gustafson, Senthold Asseng, Claudio O. Stöckle, John Kruse, Chuang Zhao, Pon Intrapapong, Marty D Matlock, Greg Thoma

**Affiliations:** a Ralph E. Martin Department of Chemical Engineering, University of Arkansas, Fayetteville, AR 72701, USA; b Agriculture & Food Systems Institute, Washington, DC 20005, USA; c Agricultural and Biological Engineering Department, University of Florida, Gainesville, FL 32611, USA; d Department of Biological Systems Engineering, Washington State University, Pullman, WA 99164-6120, USA; e World Agricultural Economic and Environmental Services, LLC, 3215 S. Providence Rd, Suite 3 Columbia, MO 65203, USA; f Department of Biological and Agricultural Engineering, University of Arkansas, Fayetteville, AR 72701, USA

**Keywords:** Life cycle assessment, Life cycle inventory, Fruits and vegetables, Processed products, Sustainability

## Abstract

This article elaborates on the life cycle assessment (LCA) protocol designed for formulating the life cycle inventories (LCIs) of fruit and vegetable (F&V) supply chains. As a set of case studies, it presents the LCI data of the processed vegetable products, (a) potato: chips, frozen-fries, and dehydrated flakes, and (b) tomato-pasta sauce. The data can support to undertake life cycle impact assessment (LCIA) of food commodities in a “cradle to grave” approach. An integrated F&V supply chain LCA model is constructed, which combined three components of the supply chain: farming system, post-harvest system (processing until the consumption) and bio-waste handling system. We have used numbers of crop models to calculate the crop yields, crop nutrient uptake, and irrigation water requirements, which are largely influenced by the local agro-climatic parameters of the selected crop reporting districts (CRDs) of the United States. For the farming system, LCI information, as shown in the data are averaged from the respective CRDs. LCI data for the post-harvest stages are based on available information from the relevant processing plants and the engineering estimates. The article also briefly presents the assumptions made for evaluating future crop production scenarios. Future scenarios integrate the impact of climate change on the future productivity and evaluate the effect of adaptation measures and technological advancement on the crop yield. The provided data are important to understand the characteristics of the food supply chain, and their relationships with the life cycle environmental impacts. The data can also support to formulate potential environmental mitigation and adaptation measures in the food supply chain mainly to cope with the adverse impact of climate change.

## Specifications Table

**Table:** 

Subject	Agriculture Science, Environmental science; Food Science
Specific subject area	Life Cycle Inventory and Life Cycle Assessment Modelling of Fruit and Vegetable supply chains
Type of data	Tables, figures and process descriptions
How data were acquired	• Ensembled data computed from the various mechanistic crop models were used for the evaluation. For potatoes, it constituted: SIMPLE, CropSyst, LINTUL-POTATO-DSS, EPIC and DSSAT-Substor-Potato); for tomato (SIMPLE, CropSyst, and DSSAT CSM-CROPGRO-tomato). Both constituted one statistical model under RCP8.5 scenario • Crop models were used to simulate the crop yields, crop nutrient uptakes and irrigation water in future climatic scenarios • Life cycle inventory for post-harvest stages were based on data available from a processing plant and through engineering estimates • Emissions calculation based on the World Food LCA Database, IPCC GHG emission protocol and Ecoinvent LCIA guidelines
Data format	Raw and analyzed
Parameters for data collection	Crop yields were initially simulated on dry matter basis, and later evaluated for the harvested moisture content (described in the method sections); emissions were computed after considering the established Nitrogen and GHG emissions protocols; reference flows of raw materials are calculated representing the functional unit of the assessment (i.e., 1 kg product consumed at consumer stage). All the parameters and assumptions made for estimating the losses and emissions during the production, processing and handling of the main products and the waste are also detailed in the Data
Description of data collection	The process of data collection constituted use of mechanistic models, expert consultations and based on engineering estimates. The presented data describe key characteristics of F&V supply chains. Data are also cross verified with other literature, whenever they are available
Data source location	United States (US) Crop Reporting Districts which account for >80% of F&V crop production in the US
Data accessibility	All the related data are within the article and detailed supplementary information is also provided
Related research article	Parajuli, R., Matlock, M. D., & Thoma, G. [1]. Cradle to Grave Environmental Impact Evaluation of the Consumption of Potato and Tomato Products. Science of The Total Environment, 143662. 10.1016/j.scitotenv.2020.143662

## Value of the Data

• The data provide a comprehensive life cycle inventory information of the processed potato and tomato products and the supply chain.
• The data can also assist to understand the methodological background for evaluating the fresh market products.
• The data cover the critical components of the supply chain and can also assist to evaluate the environmental hotspots of various fruits and vegetable supply chain.
• Physical quantities of different raw materials, as reported in the data, also support to undertake economic evaluation of the supply chain.
• The data can be beneficial to different stakeholders associated to food supply chain, such as farmers, processors, retailers, supermarkets, policymakers and LCA practitioners. It can support to formulate and implementenvironmental mitigation and adaptation strategies to investigate for sustainable food supply chain.
• LCA practitioners, academicians, students etc., can have thorough understanding on the characteristics of food supply chain, hence can use these data to evaluate environmental footprints of various food commodities and compare with similar studies*.*

## 1 Data Description

Climate change is one of the major challenges to the agriculture sector [2], which is itself a major source of the greenhouse gases (GHG's) that contribute to climate change. Depending on the types of crops and agro-climatic settings, both the quantity and quality of foods produced within the agriculture sector are impacted by the changing climate [3]. Other adverse impacts include threats to current crop protection strategies, primarily due to pest infestations, and stresses on crop-water and crop-nutrient demand [4].

In this context a multidisciplinary project was initiated in the United States (US) which is focused on evaluating the productivity, resilience, and sustainability of fruit and vegetable (F&V) supply chains [5]. Among the different components of the project: crop modeling, economic modeling, research and extension, the project also includes the use of life cycle assessment (LCA) method to evaluate the environmental footprints of the F&V supply chains. LCA is a widely used tool for evaluating environmental footprints of different production systems/processes [6].

This data article details on the LCA Protocol, illustrating the information of raw materials that are generally used in producing and consuming the processed foods. The presented data are expected to be useful reference materials for conducting life cycle impact assessment (LCIA) of different types of F&V products. At the current level, this protocol describes methods to be used for LCA modeling of three types of processed potato products (chips, frozen-fries and dehydrated) and a tomato-based pasta sauce. It also considers impact of current (year 2017) and future climatic stresses (years 2030 and 2050) on the farm productivity of potato and tomato crops (detailed in section 5).

## 2 Materials and Methods

### 2.1 LCA modelling components

**Fig 1:**
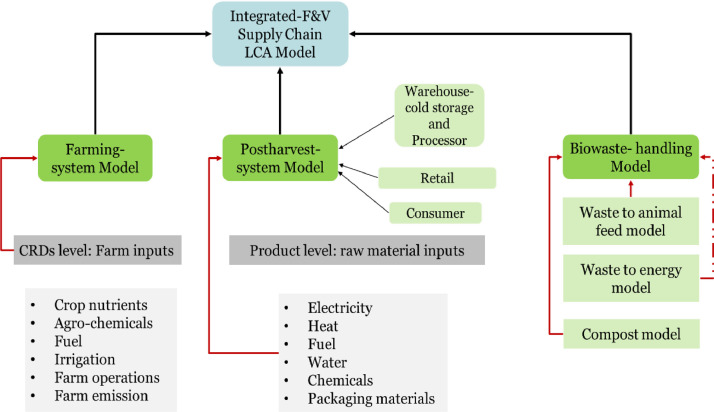
The integrated LCA F&V supply chain model, showing the three components: Farming system model, Postharvest system model, and Biowaste handling model. Warehouse/storage is only considered for the potato supply chain.

The LCA model of the F&V supply chain consists of 3 main components: (i) Farming system model; (ii) Postharvest system model; and (iii) Biowaste handling model. These three components are combined to form an Integrated-F&V supply chain model (see Fig. 1). Each component of the model is described in greater detail in the following sections. In brief, the first component is the farming system model, which represents the production of the selected crops in the selected Crop Reporting Districts (CRDs) [7]. The farm system model supplies the major input to the second component of the integrated model. The subsequent stages of the supply chain include the processor (with warehouse-storage, in the case of potatoes), retailer/supermarket and consumer. The third component describes the different methods considered for biowaste (food waste) handling that is generated across the supply chain. Section 2.2 describes the LCA specification for the baseline scenario. Biowaste handling is described in Section 3.

### 2.2 LCA specifications

#### 2.2.1 System boundaries and functional unit

**Fig 2:**
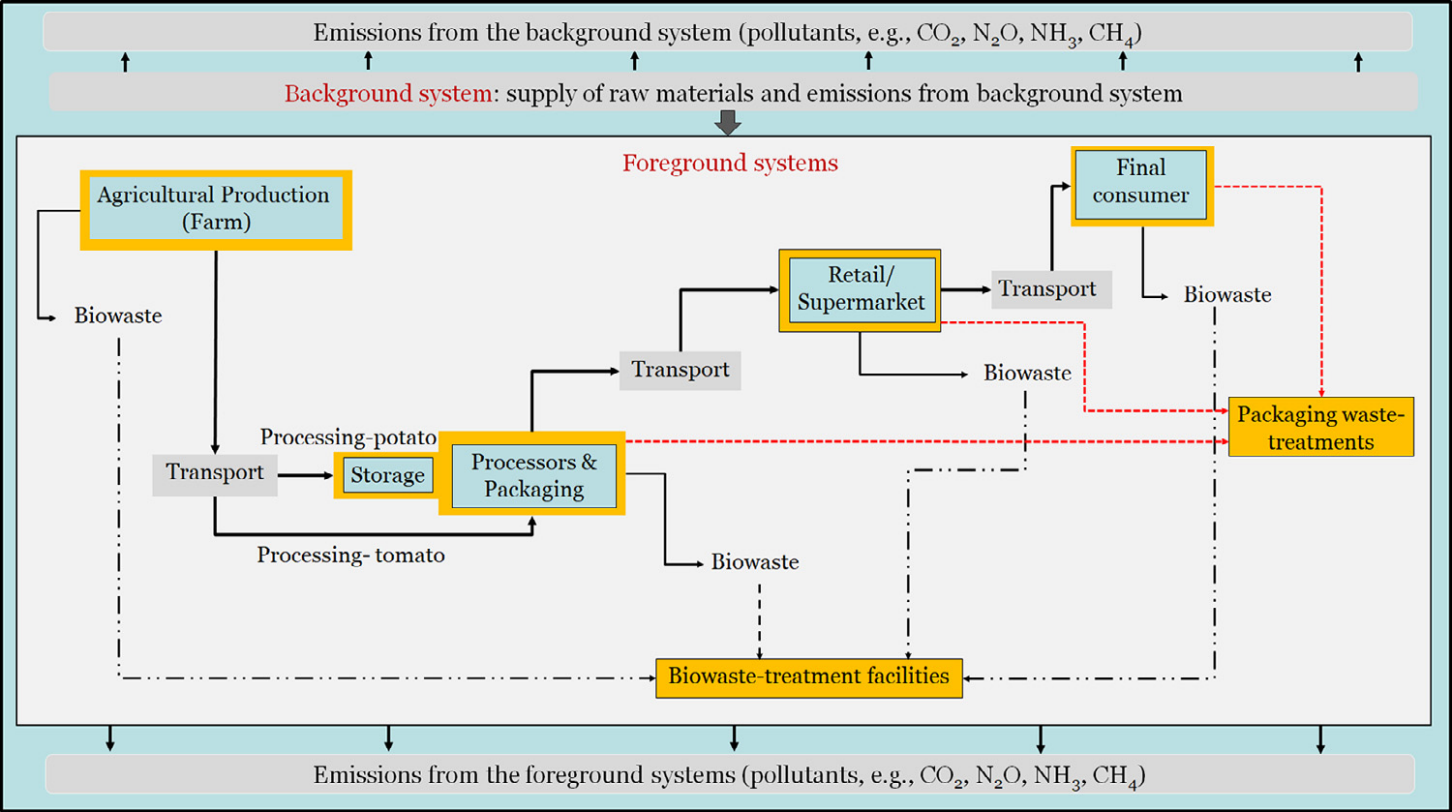
Overall schematic description of F&V supply chains, explicitly showing the Background and Foreground systems. Processing potatoes are assumed to be stored at the processor.

The system boundary defined for the F&V supply chain is presented in Fig 2. This consists of handling the reference flows in a “cradle to grave” perspective (farm to consumer, including waste). Reference flows (see Table 1) are the quantity of the outputs from individual unit processes that constitute the product system fulfilling the functional unit (FU). With this data article, the LCA practitioners may have flexibility to evaluate the environmental footprints for any system boundary/stage of the supply chain. The defined FU is 1 kg product, eaten at consumer, for both potato products and tomato-pasta sauce. It should be noted that the reference flow of the raw crops accounted the loss fractions occuring at each stage of the supply chain (Section 2.3.1).

**Table 1:** Illustration of reference flows of raw products yielding the Functional Unit (1 kg of processed food product).

		Potato products			
	Unit	Potato-chips	Potato-frozen fries	Potato-dehydrated	Tomato product Tomato-pasta sauce
Functional Unit (m)^a^	kg	1	1	1	1
Reference flows					
Products prepared at consumer (m_c_)^b^	kg	1.22	1.22	1.22	1.22
Supply at Retailer (m_r_)^c^	kg	1.30	1.3	1.3	1.3
Supply at processor (m_p_)^d^	kg	1.90	2.12	1.94	5.65
Farm supply (m_f_)^e^	kg	1.94	2.16	1.98	5.76

Assumptions:

a Functional Unit (FU), as the final weight, actually eaten at consumer. FU = 1 kg product (98% total solids-potato) and 1 kg product (31% total soluble solids-tomato) (see Section 2.5.1).

b Product, eaten at consumer (m_c_) = 1 kg (i.e. FU). The reference flow also accounted the losses at the consumer stage.

c Supply at retailer (m_r_) = Final process products packed at processor with respect to the FU (m_p_) * % losses at retailer (see Appendix 2).

d Supply at processor (m_p_) = (m_r_ /product recovery). For the product recoveries (Tables-5-6). Detailed in Appendix 10. Shrink losses at store = m_f_/(100%-% shrink losses). Losses = 2.07% (reported range is 0-4.9%) [18] (Appendix-2). Shrink losses are also considered for tomatoes, despite they do not undergo storage for a longer time.

e Farm supply (m_f_) = m_p_ /(100%-% losses between farm and retail). See Appendix 2.

Fig 2 shows the system boundary of the reference flow of materials and the emitting sources in the background and foreground systems. The background system represents all the related upstream activities that supply the required raw materials (e.g., fertilizers, fuels, agricultural implements, and packaging materials) to the main system being investigated. Detailed LCIs of the assumed raw materials (at the background level) are adopted from the Ecoinvent LCA database, v3.6 [8]. The foreground system is the main system for which a life cycle assessment is performed.

In the LCI modeling process, raw potatoes received from farm are assumed to be stored in a controlled (refrigerated) environment to maintain a year-round-supply of potatoes. The storage facility was assumed to be within the processor premises. In the case of tomatoes, larger processing facilities generally contract with specific farmers and buy bulk amounts of the raw product and lead to the respective processing lines (to prevent losses and damage in between due to storage). Therefore, before processor, no storage facility was considered in the tomato supply chain (Fig 2). After processing, the final products are delivered to a retail market (supermarket), which then finally reach to consumers. In the case of fresh market, a wholesale storage stage is often involved so that fresh products are delivered to retail outlets.

In the current data since we have considered multiple crops producing states across the U.S (see Appendix 1), it necessitated assuming suitable transportation distance. The average distance reported between food processing facilities and farms was 109-560 km [9,10]. For tomatoes, 111-160 km is a suitable distance, suggested for reducing the cost associated with logistics [9,10], while for potatoes it ranged between 240 to 560 km. Hence, to cover the wide range of crops (considering transport from farm to processing facilities, in the selected CRDs), we assumed 267 km (averaged from potatoes and tomatoes). Likewise, the distance from processor to retail was assumed at 1200 km. The distance was calculated considering the average distance from the available potato and tomato processing facilities in the US to Kansas City, Missouri (MO), assuming the MO a mid-point of the US. Hence after considering the distances: farm to processor and processor to retail, the total distance was 1467 km.

Regarding the transportation mode, one of the studies prepared in late 1970’s suggested that in the US approximately 60% of food and related products were transported from the farm by truck and the remaining 40 percent by rail, as argued in Pirog et al. [11]. However, Pirog et al. [11] further suggested that in past 25 years (from the 1970s), with the improved road infrastructures in the US, the amount of food transported by truck has increased dramatically. For example, nearly 93% fresh produce transported between cities in the US was moved by truck. We have thus assumed that transportation mode from processor to retail was truck. For frozen fries, transportation involved a refrigerated truck. For the processing tomatoes, transport via train was also involved between the processing units (see Section 2.5.2).

#### 2.2.2 Impact categories and impact assessment methods

The choice of impact categories and impact assessment methods is generally governed by the scope of a study. ISO (2006) [12] also suggests that the choice should be based on the specific requirements of the LCA practitioner for meeting the objective of a study [13]. To analyze and check the LCIs with respect to the potential environment impacts, this protocol used three impact categories, expressed per FU are: Global Warming Potential (GWP_100_) (in kg CO_2_ eq), Water consumption (m^3^ eq) and land use (m^2^-a). However, this does not limit the evaluation of other potential environmental impact categories. The outputs of such are also discussed in Gustafson et al. [14].

#### 2.2.3 Product and co(products) handling approaches

Many production systems generate multiple products with various functions and services. Handling of multi-functional processes in LCA involves a choice among different approaches, such as sub-division, system expansion and allocation [15]. This often occurs in the food processing industry, where processing plants are built with multiple processing lines, which generate arrays of products (e.g., raw potato processed to frozen fries, chips, dehydrated products; and tomato to paste, diced, sauces etc.). In such cases, as suggested in Ekvall and Weidema [16] and European Commission (2010) [13], physical causal relationships were applied to the extent possible. In the current protocol, it is assumed that even if the quantity of frozen fries produced from a processor changes, the quantity of potato-chips is not significantly affected. Hence, from the total annual raw materials consumed in an ideal processing plant, we quantified the amount for each product line. The method for portioning the raw crops incorporated the use of a product recovery factor (e.g., yield of chips and fries from the total raw potato received at processor gate) (see Section 2.5.2). At the processor, each product produced from the potato processing line was treated as the “main product” (see Section 2.5.1). Co-products, including waste/losses are handled through a system expansion approach of LCA. In system expansion, particularly in a consequential LCA (CLCA) approach, related unit processes/activities of a product system are expected to change because of a change in demand for the functional unit [17]. Hence, whenever the co-products have certain functionality (e.g., feed values, energy values and fertilizer values), the consequences of their production on the affected market were accounted. It is handled in the form of substitution of the available conventional products in the market. A detailed description of the (co)product and biowaste handling approach is described in Section 3.

### 2.3 Integrated LCA model components descriptions

#### 2.3.1 Reference flows

Reference flows of raw materials in each stage of the supply chain were calculated with respect to the FU (i.e., 1 kg processed product). The reference flow is the amount of each product category that passes the respective stages of the supply chain to provide the stated functional unit, hence it accounts the losses/wastes. As an example, to consume 1 kg of frozen fries, 1.22 kg of the product is to be prepared accounting for consumer waste of 18%. The waste proportion at different stages of the supply chain is presented in Appendix 2. So, to provide the FU of the potato-frozen fries, the required quantity of raw potato that must be supplied from the farm is estimated to be 2.16 kg (Table 1), including all supply chain losses.

### 2.4 Farming system model and LCIs

#### 2.4.1 Crop production and supply at farm gate

Crop Reporting Districts (CRDs) producing most of the targeted crops (>80%) in the US [7] were included in the farming system modeling. Potatoes (processing) included 26 CRDs and 13 CRDs were included for tomatoes (processing) (see Appendix 1). The farming system model included the effects of technology advancement and climate trends while forecasting the future crop yields Gustafson et al. [14]. The outputs from the crop modeling [7], particularly the current and the future estimates of crop yield, crop water use and fertilizer (NPK) requirements were used in the LCA model (see Appendix 1.b). For potato, five mechanistic models (SIMPLE, CropSyst, LINTUL-POTATO-DSS, EPIC and DSSAT-Substor-Potato) [19],[96],[20],[21] were used. Likewise, for tomatoes three crop models (SIMPLE, CropSyst, and DSSAT CSM-CROPGRO-tomato) [19],[96],[22] were used. Both crops additionally used one statistical model model [23] under RCP8.5 scenario. Ensemble of the crop modelling outputs were finally considered for LCIA. The CropSyst simulated the water demand for the selected crops [7]. Detailed description on the integrated method used for crop modeling is further elaborated in Gustafson et al. [14].

Raw material inputs were primarily based on the crop-specific Enterprise Budgets, published by State Extension Services [24], [25], [26] and other sources [27]. Apart from these data sources, USDA/NASS crop production survey data and other sources (as noted elsewhere in this protocol) were also used to fill data gaps. Table 2 shows an example of the raw materials inputs and represent the average production and raw material inputs across the selected CRDs (Appendix-1). CRD-wise data computed using this LCA Protocol can be found in the supporting information of Parajuli R et al. [1]

**Table 2:** Reference flows of raw materials to produce potato and tomato products. Values shown are with respect to the Functional Unit (1 kg processed food product). Values shown represent average production of the selected CRDs. Standard deviations (SD) in the reference flow are shown in parenthesis.

		Potato products			Tomato product
	Units	Potato-chips	Potato-frozen fries	Potato-dehydrated	Tomato-pasta sauce
Functional Unit^a^	kg	1	1	1	1
Farm inputs^b^					
Agro-chemicals					
N	kg	1.29*10^−2^ (4.65*10^−18^)	1.44*10^−2^ (1.51*10^−18^)	1.31*10^−2^ (3.18 *10^−18^)	8.46*10^−3^ (1.42*10^−18^)
P_2_O_5_	kg	1.65*10^−3^ (8.58*10^−19^)	1.83*10^−3^ (4.2*10^−19^)	1.68*10^−3^ (7.27*10^−19^)	1.44*10^−2^ (1.52*10^−3^)
K_2_O	kg	1.36*10^−2^ (8.39*10^−4^)	1.52*10^−2^ (9.34*10^−4^)	1.39*10^−2^ (8.54*10^−4^)	1.30*10^−2^ (1.37*10^−3^)
Lime	kg	2.48*10^−2^ (7.54*10^−3^)	2.76*10^−2^ (8.4*10^−3^)	2.53*10^−2^ (7.67*10^−3^)	4.22*10^−2^ (2.16*10^−2^)
Sulfur	kg	3.88*10^−3^ (1.2*10^−3^)	4.32*10^−3^ (1.31*10^−3^)	3.95*10^−3^ (1.2*10^−3^)	2.96*10^−3^ (6.98*10^−4^)
Zinc	kg	1.66*10^−4^ (5.05*10^−5^)	1.85*10^−4^ (5.62*10^−5^)	1.69*10^−4^ (5.14*10^−5^)	2.99*10^−7^ (7.04*10^−8^)
Magnesium	kg	2.61*10^−4^ (7.94*10^−5^)	2.91*10^−4^ (8.84*10^−5^)	2.66*10^−4^ (8.08*10^−5^)	-
Gypsum	kg	6.4*10^−3^ (1.94*10^−3^)	7.1*10^−3^ (2.17*10^−3^)	6.5*10^−3^ (1.98*10^−3^)	8.27*10^−2^ (1.27*10^−17^)
Boron	kg	5.38*10^−5^ (1.64*10^−5^)	5.99*10^−5^ (1.82*10^−5^)	5.48*10^−5^ (1.66*10^−5^)	-
Total Pesticide (a.is.)	kg	8.81*10^−4^ (4.58*10^−19^)	9.82*10^−4^ (6.45*10^−19^)	8.97*10^−4^ (5.63*10^−19^)	3.39*10^−3^ (7.75*10^−4^)
Farm operations^c^	Fuel (pls see the texts, in Section 2.4.1)				
Irrigation (water)	m^3^	1.11*10^−1^ (7.04*10^−2^)	1.24*10^−1^ (7.84*10^−2^)	1.13*10^−1^ (7.17*10^−2^)	1.48*10^−1^ (5.7*10^−2^)
Transport (farm to farm store)^d^	t-km	7.39*10^−3^ (2.25*10^−3^)	8.06*10-^3^ (2.45*10^−3^)	7.37*10^−3^ (2.24*10^−3^)	2.88*10^−2^ (4.48*10^−18^)
Farm implements^c^					
Farm outputs					
Harvested weight required^e^	*kg*	1.94 (1.13*10^−15^)	2.16 (9.06*10^−16^)	1.98 (6.79*10^−16^)	5.76 (5.9*10^−17^)
*Waste (at farm-retail)*^f^	kg	4.47*10^−1^ (6.79*10^−3^)	4.98*10^−1^ (7.56*10^−3^)	4.55*10^−1^ (6.91*10^−3^)	1.26 (5.48*10^−16^)
Emissions	For N-emissions, see Table 3				
CO_2_ (Lime + Urea)^g^	kg	1.4*10^−2^ (3.32*10^−3^)	1.52*10^−2^ (3.69*10^−3^)	1.39*10^−2^ (3.38*10^−3^)	6.67*10^−1^ (3.07*10^−4^)

Assumptions:

a See Table 1.

b See texts (Section 2.4.1).

c Farm implements for potatoes and tomatoes are based on Ecoinvent v3.6 for the US potato production.

d Assumptions for the transport distance, in tons-kilometer (tkm) shown in Appendix -8.

e Yield included losses at farm (loss %, see Appendix 2). Harvested yields are with respect to the FU. Potato yields per ha, averaged at 56.9 t (max: 91.75 t, min: 36.5 t); tomato, averaged at *101 t (max: 112 t, min: 80 t).*

f Waste at farm to retail was assumed (see Appendix 2). The harvested yield is waste corrected.

g Emission factor based on [29]. See Section 2.4.2.

Background data for the farm implements was adopted from Ecoinvent v3.6 [8]. CRD-specific raw material inputs (mainly for pesticides and other crop micro-nutrients) were not available for all the CRDs, therefore, data reported in the Crop Budgets Reports and USDA/ERS for the states California, Florida, Idaho and Washington were selected as reference. Such reference data sources were used for estimating pesticide inputs (total active ingredients) [28] and crop micro-nutrients (e.g., zinc, boron, copper, as relevant) [24], [25], [26]. The reference data were extrapolated proportionately with respect to the crop yields in the respective CRDs (see the LCIs in Table 2 and yields of CRDs, as shown in Appendix-1).

The amount of the crop nutrients (NPK) applied were back-calculated by utilizing the nutrient uptake results reported for the respective crops in the Crop Modeling protocol [7]. Nutrients uptakes in the potato-tubers and tomato fruits were used for calculating the fertilizer inputs. Total crop biomass (including the harvested biomass, such as, fruits and tubers, along with non-harvestable biomass, both above- and below-ground) was used in the crop modeling protocol to estimate the nutrient uptake as a fraction of crop yield (nutrient harvest indices) [7]. For the estimation of fertilizer inputs, the yield-based nutrient uptakes were considered along with the following assumptions:

• NHI (nitrogen harvest index) = This is defined as the ratio of N in the harvested crop item (in this case, potato tuber or tomato “fruit”) to the total crop biomass N uptake. It is generally in the range of 60-75% for potato and tomato [30], [31], [32], [33], [34]. In this protocol, the calculated NHI for potato was 67% (ranged from 45% to 88% across the CRDs) and tomato was 53% (ranged from 42% to 58% across the CRDs) [7]. This can be used whenever crop nutrient uptakes related to the yield (fruits or tubers) are to be calculated from the total biomass nutrient uptakes.
• NUE (nitrogen uptake efficiency) = It is considered as the amount of N accumulated in the plant per unit of harvested biomass (i.e. yields) [35]. For example, the NUE for the potato and tomato crops is 0.36-0.64 [34,^36^,37] and 0.53-0.7 respectively [38], depending on the fertigation practices. For potato the NUE was assumed as 0.5 (assuming central pivot irrigation) [30], [31], [32], [33], [34]. In the case of tomato, since drip irrigation was assumed, the NUE was set at 0.68.
• Phosphorous and Potassium uptake was based on the Crop Model. P-uptake efficiency was assumed at 90%, whilst for K fertilizer it was assumed to be 100% [39]. P and K were converted to P_2_O5 and K_2_O using their respective molar ratios.

Emissions related to the production of the applied crop nutrients and pesticides were adopted from Ecoinvent v3.6 [8]. Application rates for the pesticides, in terms of active ingredient (a.is.), were taken after reviewing variety of sources including the USDA/NASS and available Crop Budget Reports. For potatoes, average a.is., were calculated considering the application rate and harvested areas in the different potato producing states of the U.S., in the year 2016. Likewise, for tomatoes, pesticides application rate reported for California (for year 2016) was assumed. Due to unavailability of data, for the remaining CRDs producing processing tomatoes (other than California), average application rates of the pesticides reported for producing fresh tomatoes in the US [40] was assumed (see Appendix 1. c-e).

Irrigation water requirements were based on the crop model [7]. Central pivot and drip irrigation systems were assumed for potatoes and tomatoes, respectively. LCIs for drip irrigation infrastructure are shown in Appendix 4. Finally, the harvested, raw F&V products are assumed to be directly delivered to the processor by truck (described in Section 2.5).

#### 2.4.2 Farm emission calculations

Soil carbon accumulation was not included, as the selected crops are not expected to substantially affect soil carbon changes during direct land use occupation [41]. CO_2_-binding elements (expressed as CO_2_ emissions to air) were set at 1.55 and 1.65 kg CO_2_-eq per kg DM potato and tomato crops [42]. GHG emissions due to applications of lime and urea were assumed to be 0.44 kg CO_2_-eq per kg CaCO_3_ (limestone) and 1.57 kg CO_2_-eq per kg Urea [29].

A partial nitrogen balance approach [43], [44], [45], [46] was used to quantify the total N inputs and outputs, including field losses (Table 3). For N inputs, the contributions from various sources were (i) synthetic fertilizer, (ii) compost (depending on the waste handling scenarios, discussed in Section 3), (iii) atmospheric deposition (ranges 1-2 kg per ha per season), (iv) N available from seeds (ranges 3-4 kg N per ha) [34]. Additional N-contributing sources reported with higher uncertainty were excluded [47,48]. The excluded components were nitrogen mineralization from crop residues and soil organic matter due to high uncertainty [34,41]. Likewise, N present in irrigation water was excluded due to uncertainties with the seasonal fluctuations in N concentrations in the water sources across the selected CRDs [34,49]. For compost, equivalent fertilizer efficiency to the synthetic fertilizers was assumed at 15% [50]. The amount of nutrient available from compost was assumed to substitute synthetic fertilizer (detailed in section 3.1). Direct and indirect nitrous-oxide emissions (N_2_O-N) were based on standard emissions factors [29]. Factors assumed for NH_3_ emission from N fertilizer were based on [42]. Denitrification losses were based on other studies [34,51] (see Table 3). NOx emission was calculated after IPCC (2006) [29], following the steps (i) volatilisation from synthetic fertilizer = 0.1 kg (NH_3_-N+NOx-N) per kg N-fertilizer, (ii) from the step , NH_3_-N (e.g., shown in Table 3) was deducted to obtain NOx-N, and (iii) finally the converted NOx-N to NO_2_- was used in LCIA. Phosphorus losses to ground water and river were calculated following the Nemecek et al. [42]. For pesticides, it was assumed that 100% of the applied active ingredients are emitted to soil [8].

**Table 3:** Calculations of emissions. N emissions based on partial N balance method. Values are shown with respect to the Functional Unit. Values shown represent average production across the selected CRDs. Standard deviations (SD) in the reference flow are shown in parenthesis.

		Potato products			
	Units^1^	Potato-chips	Potato-frozen fries	Potato-dehydrated	Tomato product Tomato-pasta sauce
Functional Unit	kg	1	1	1	1
N-emissions calculations					
Total-N input^a^	kg	1.32*10^−2^ (7.4*10^−3^)	1.47*10^−2^ (8.24*10^−5^)	1.34*10^−2^ (7.53*10^−5^)	8.87*10^−3^ (9.8*10^−5^)
N-uptake^b^	kg	6.5*10^−3^ (2.3*10^−18^)	7.19*10^−3^ (7.6*10^−19^)	6.57*10^−3^ (1.6*10^−18^)	5.75*10^−3^ (1.32*10^−18^)
Field balance^c^	kg	6.7*10^−3^ (7.4*10^−5^)	7.19*10^−2^ (8.24*10^−5^)	6.82*10^−2^ (7.53*10^−5^)	3.12*10^−3^ (9.8*10^−5^)
N-Losses	kg	4.88*10^−4^	5.32*10^−4^	4.87*10^−4^	3.13*10^−4^
*NH_3_-N*	*kg*	2.58*10^*−4*^ (1.25*10^*−19*^*)*	2.88*10^*−4*^ (1.35*10^*−19*^*)*	2.63*10^*−4*^ (1.7*10^*−19*^*)*	1.7*10^*−4*^ (2.6*10^*−20*^*)*
*NO_x_-N*	*kg*	9.04*10^−5^ (1.12*10^*−20*^*)*	1.01*10^−4^ (2.2*10^*−19*^*)*	9.2*10^−5^ (2.2*10^*−20*^*)*	5.92*10^*−5*^ (6.8*10^*−21*^*)*
*Denitrification*	*kg*	1.29*10^−4^ (6.25*10^*−20*^*)*	1.44*10^−4^ (6.8*10^*−20*^*)*	1.31*10^−4^ (8.4*10^*−20*^*)*	8.46*10^*−5*^ (1.3*10^*−20*^*)*
Total N_2_O-N	kg	1.79*10^−4^ (5.75*10^−7^)	2.0*10^−4^ (6.2*10^−7^)	1.83*10^−4^ (5.65*10^−7^)	1.08*10^−4^ (7.33*10^−7^)
(a) Calculation for N_2_O (direct)	kg	1.29*10^−4^	1.44*10^−4^	1.31*10^−4^	8.46*10^−5^
*N_2_O-N* *(N-synthetic application)*^d^	*kg*	1.29*10^*−4*^	1.44*10^*−4*^	1.31*10^*−4*^	8.46*10^*−5*^
(b) Calculation steps for N_2_O (indirect)	kg	5.02*10^−5^	5.59*10^−5^	5.11*10^−5^	2.33*10^−5^
*NH_3_-N (from N-synth application)*^e^	*kg*	2.58*10^*−4*^	2.88*10^*−4*^	2.63*10^*−4*^	1.7*10^*−4*^
*NO_x_-N (from N-synth application)*	*kg*	9.04*10^*−5*^	1.01*10^*−4*^	9.2*10^*−5*^	5.92*10^*−5*^
NO_3_-N (potential leaching)^f^	kg	6.23*10^−3^ (7.4*10^−5^)	6.93*10^−3^ (8.24*10^−5^)	6.34*10^−3^ (7.53*10^−5^)	2.81*10^−3^ (9.77*10^−5^)
Phosphorous emissions^g^					
Phosphate (ground water)	kg	7.7*10^−11^ (4.56*10^−11^)	8.58*10^−11^ (5.08*10^−11^)	7.84*10^−11^ (4.6*10^−11^)	4.49*10^−11^ (2.43*10^−11^)
Phosphorous (river)	kg	3.82*10^−11^ (2.26*10^−11^)	4.25*10^−11^ (2.52*10^−11^)	3.89*10^−11^ (2.3*10^−11^)	3.2*10^−10^ (1.73*10^−10^)
Phosphate (river)	kg	2.09*10^−10^ (1.19*10^−10^)	2.33*10^−10^ (1.33*10^−10^)	2.13*10^−10^ (1.21*10^−10^)	5.56*10^−10^ (2.28*10^−10^)

Assumptions:

1 Resource inputs and outputs represent the average production calculated for the selected CRDs. The list of selected CRDs is shown in SI (Appendix 1).

a Total N inputs, see texts (for the contributing sources).

b N uptakes, from the Crop Models [7].

c N balance = N input minus N losses.

d 0.01*F_SN_[29].

e 0.02*F_SN_[29].

f 0.0075*NO_3_-N+ 0.01*(NH_3_-N + NOx-N) [29].

g P emissions based on Nemecek et al. [42].

**Table 4:** LCI for the warehouse (storage) at processor. Values shown with respect to the Functional Unit. For tomato, storage was not required, only the distance travelled is mentioned for the reference flow.

		Potato products			
	Units	Potato- chips	Potato-frozen fries	Potato-dehydrated	Tomato productTomato-pasta sauce
Functional Unit	kg	1	1	1	1
Inputs					
Crops a	kg	1.94	2.16	1.98	5.76
Refrigerated storage b					
Electricity consumption	kWh	2.62*10^−1^	2.92*10^−1^	2.67*10^−1^	-
Transport (farm to processor)^c^	t-km	5.2*10^−1^	5.78*10^−1^	5.28*10^−1^	1.54
Outputs					
Crops d	kg	1.90	2.12	1.94	5.65
Losses					
Shrink loss	kg	4.01*10^−2^	4.5*10^−2^	4.1*10^−2^	1.2*10^−1^

Assumptions:

a From Table 3, product output with respect to the FU. Harvested weight was calculated considering the losses (between the farm and retail).

b Refrigeration capacity, see Appendix 6. Infrastructure lifetime of 15 years [58].

c Transport distance (one way) presented in Appendix 8.

d Product output after accounting for shrinkage losses (2.05%). Losses are calculated based on the reports [59], [60], [61], [62] (Appendix 2).

### 2.5 Post-harvest components

#### 2.5.1 Processor

a Storage

Potatoes are assumed to be stored at the processor in a refrigerated environment. Tomatoes are delivered from farms and received directly at processor gates for immediate processing (see Section 2.5.1.b). For storing raw potatoes, the cooling load calculations were based on the optimum product cooling temperature of the selected F&V crops [52], and the most severe conditions expected during the storage of the products. Refrigeration capacity was calculated considering the thermal properties of the F&V products, building materials, and the packaging materials. The sizing of the storage facilities and properties of the insulating materials were based on the methods reported in Boyette et al. [53] and other data sources [54], [55], [56], [57].

The total heat that the refrigeration system has to remove from the storage space included heat loss from the refrigerated space: conduction loss through walls, roof, floors; field heat (heat from the products dissipated in the storage); heat of respiration (heat generated as a natural by-product respiration and service load (heat from the equipment, lights, people etc., assumed at 10% of the total heat load) (see Appendix 6).

a Potato processing lines

The raw potatoes are assumed to be handled in a multiple processing lines at the processor. A detailed LCI for the processor is shown in Appendices 9-10. A summarized LCI is shown in Table 5. Three processing lines were assumed for potato: chips, frozen fries and dehydrated (Appendix 10). Hourly processing capacity reported by [63] was used to sub-divide the reference flows across the three processing lines. The method for subdivision incorporated the use of the product recovery factor (i.e., yield of chips, fries and dehydrated products from the raw potato received at processor gate), which is calculated from [63] (see Appendix 10). For the drying steps (particularly for dehydrated product), it also followed the basic assumptions reported in the studies [64], [65], [66].

**Table 5:** Processing of raw potatoes to produce selected products. Values shown with respect to the Functional Unit.

		Potato products		
Processors	Units	Potato-chips	Potato-frozen fries	Potato-dehydrated
Functional Unit	kg	1	1	1
Inputs				
Crop^a^	kg	1.90	2.12	1.94
Other raw materials inputs				
Total electricity b	kWh	5.52*10^−3^	9.21*10^−1^	1.6*10^−1^
Heat consumption, natural gas^b^	MJ	2.89*10^−2^	3.22*10^−2^	2.94*10^−2^
Water use^b^	kg	3.48	5.67	5.54
Salt^b^	kg	3.95*10^−2^	-	-
Oil^b^	kg	6.42*10^−2^	3.46*10^−2^	-
Antioxidant^b^^,*^	kg	9.02*10^−4^	-	-
Fatty acid^b^	kg	9.02*10^−4^	-	-
Packaging material^c^				
LDPE bags^∆^	kg	2.9*10^−8^	4.1*10^−3^	4.1*10^−3^
Cardboard boxes^±^	kg	5.6*10^−2^	3*10^−2^	5.6*10^−2^
Aluminum (MOPP)	kg	1.2*10^−15^	-	-
Outputs				
Potato products^d^	kg	1.67	1.5	1.3
By-products/waste^e^				
Total biowaste^f^	kg	5.62*10^−1^	7.48*10^−1^	5.72*10^−1^
Starch^e^	kg	2.52*10^−3^	2.0*10^−2^	1.99*10^−2^
Waste water^d^	m^3^	3.48*10^−3^	5.67*10^−3^	5.54*10^−3^
Oil waste^g^	kg	6.42*10^−3^	3.46*10^−3^	-

Assumptions:

a Crop supply from the farm and wholesales-store, after including loss at the store.

b Energy, water and other inputs (see Appendix 10). * Antioxidant used is assumed to be onions.

c Packaging material estimates: Potato chip packaging [68,69] included both oriented polypropylene (OPP) and metalized oriented polypropylene (MOPP); and OPP was assumed for Potato fries [69]. ^Δ^Plastic pouch for dehydrated potato was assumed as chips, but only the OPP (oriented polypropylene) portion [69] was assumed. References for the packaging plastics also reviewed from [70]. ^±^ Weight of the corrugated boxes considered a handling capacity of 22.5 kg. Dimensions and other parameters assumed for the corrugated box and the other packaging materials are detailed in Appendix 5. Plastic materials (as inputs) were adopted from the ecoinvent database of the LDPE plastic film.

d Product output shown with respect to the FU available at consumer (see Table 1). Product yield = 66.67% (chips), 61.35% (fries) and 67.11% (dehydrated) of the raw potato received at processor gate (output divided by input at processor). Mass include the oil (for chips and fries) and moisture content in the product.

e Starch is recovered and assumed to be sold to the market, substituting the available corn-based starch (see section 3).

f Total biowaste = peels + potato scraps (see above) + unwanted potato sorted during the destoning process (see text, section 2.5.1. b).

g See text, Appendices 8-9.

Regarding the processing steps, the first stage was the handling of the raw potato (received at processor gate), where processes such as destoning and washing take place, followed by delivering potatoes to the peeling unit. About 0.6% of the raw potato received at processor gate is removed [63] during the destoning process. Water required for washing was estimated to be 1.4 kg per kg fresh potatoes. The washed raw potatoes were then assumed to enter the peeling chamber through a conveyor. Steam (0.55 kg steam per kg raw potato) is assumed to be used to peel the product, requiring about 21 kJ fuel per kg raw potato. The peel scrap was estimated to be 24% of the weight of the raw potato received at the processor gate. The peeled potatoes are then conveyed to the slicing unit, producing about 0.71 kg sliced potato per kg raw potato, and the waste from the slicing unit was estimated to be 5% of the raw potato. The sliced potatoes are then washed, producing about 0.68 kg washed and sliced potato, and along with the by-products such as starch (about 2% of the raw potato) and wastewater. Water used for washing was estimated at 0.37 kg per kg raw potato. In the case of producing potato-frozen fries and dehydrated potato, the washed and sliced potatoes are subjected to blanching chamber, up to which they are conveyed through an automatic conveyor. Because the starch in potato slowly turns to sugar during storage, blanching helps to maintain a constant sugar level and helps to ensure that fries have a consistent texture and colour. 0.53 kg blanched potato was estimated to be produced per kg raw potato, and additionally 0.11 kg starch is produced. Sliced and blanched potatoes are then conveyed to a dewatering unit. For production of potato-chips and frozen fries, the dewatered potato is conveyed to the frying units. In the case of dehydrated-potatoes, the dewatered sliced-potatoes were conveyed to a multi-stage drying process (discussed later in this section).

**Fig 3:**
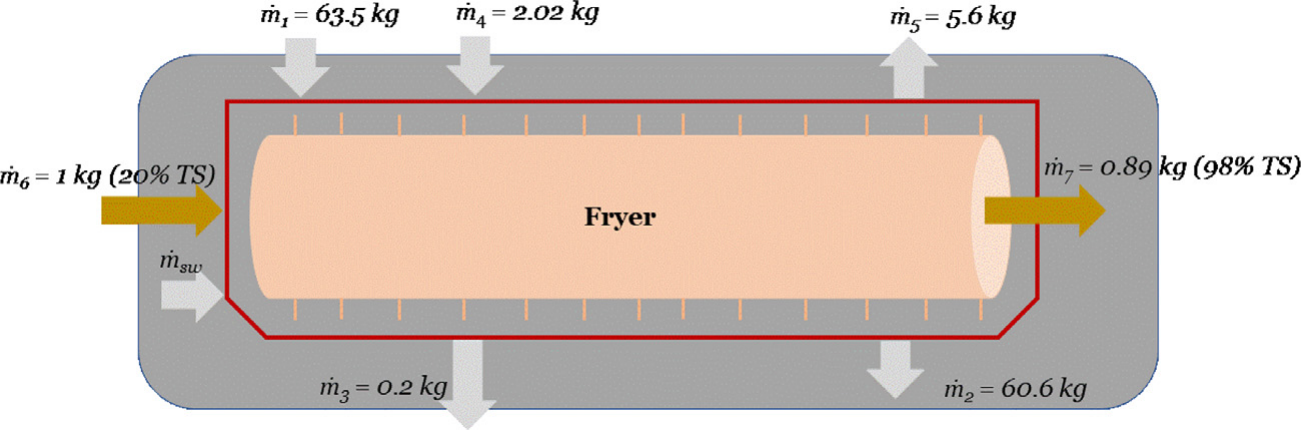
Mass flow balance for the fryer. Mass of materials (*ṁ_n_)* are shown in Table A-3. Method based on [67]. Mass flow rate per hour of the raw materials in the fryer: *ṁ_1_ = oil input, ṁ_2_* = oil return, *ṁ_3_ = fines removal, ṁ_4_* = air inflow, *ṁ_5_* = frying vapors, *ṁ_6_* = raw potato input, *ṁ_7_* = fried potatoes output. Masses are shown in Appendices 9,10.

For the products entering the fryer, mass flow analysis inside the frying unit (Fig. 3) was based on the method reported in [67] (see Appendix 9). Vegetable oil consumed by the frying units was assumed to be 0.06 and 0.035 kg per kg chips and fries respectively (Table 5). Waste oil was assumed to be treated in a waste management process (Used vegetable cooking oil, purified (Global, market for Conseq, U) [8].

In addition to the processes involved in chips and dehydrated potato, frozen fries are subjected to pre-cooling units so that the temperature of the fries (85°C after frying) is lowered to 2°C. The packed frozen fries after pre-cooling are made ready for freezing at the cold store, which is carried out in multiple stages: (i) lowering to just below freezing (-1.7 °C), (ii) and then further lowering the temperature to -18 °C. The frozen fries are then delivered to the distributor/retail, where they are stored at -18 °C, until purchased by a consumer.

In the case of dehydrated potato, the steam-cooked potatoes are dewatered and then subjected to drying. For drying, a two-stage rotary air dryer was assumed, as it is among the most common type of dryer used [64], [65], [66]. In this type of dryer, the first stage of the dryer is divided into two sections, the first at 102 °C (generally in the range 93–127 °C), and the second at 88 °C (is in the range of 71–105 °C) [64,66]. A moisture content of 6–7% for dried potato is enough for proper storage. A further extension of the storage time will require reduction of moisture content to 3–4%, which can be achieved by long-time sorption drying using sorption agents such as calcium oxide, which was not included in the current data, due to the absence of available mass balance information. Detailed resource inputs and material flows, and the calculation steps for the processing lines are reported in Appendix 10.

a Tomato processing lines

The processing of raw tomatoes to finally producing pasta sauce (with tomato+water+ ingredients) was handled in two stages: (i) the processing of raw tomato to produce tomato ingredients and (ii) processing of tomato ingredients to produce tomato-pasta sauce. From processing stage 1, the intermediate ingredients are assumed to be transported to another processing facility (where pasta sauce is produced). At the first stage of processing, the raw tomatoes are sorted, removing sediment and rocks, along with defective tomatoes. The sorted raw tomatoes, with about 5-6% total soluble solids (TSS), are then sent to the processing lines, where aseptic diced tomatoes and aseptic tomato paste are produced. After sorting, the raw tomatoes are crushed and heated to produce tomato pulp, undergoing a hot-break process [71]. The pulp is then screened in an extraction process. In the extraction process seeds and other unwanted materials are removed, which will lead to produce pure “juice consistency” liquid.

The tomato juice is then evaporated, where the incoming tomatoes (with 6% TS) are processed using steam-based heat to a paste. The next step is sterilization, where the paste is heated to 98 °C.

For producing aseptic diced tomatoes, the raw tomatoes are cleaned, sorted, and steam peeled. The peels are scrubbed and sent to the paste processing line to extract remaining tomato pulp as part of paste production. The peeled whole tomatoes are then diced and batched with tomato juice at 75% drain weight concentration. This product is then sterilized at 102 °C and cooled to ambient temperature via tube-in-shell cooling.

The product (paste or dice) is then aseptically packaged, where a sterile plastic bag is used which is then packed in a reusable plywood box. The 300-gallon capacity bag-in-box is then transported to another processing facility to produce pasta sauce.

The second stage involves processing the aseptic paste and aseptic diced tomatoes to produce the final products. For pasta sauce products, there are array of other recipes that are used, but all consist primarily of reconstituted tomato puree (tomato paste and water). The LCI related to the production of pasta sauce is shown in Table 6.

**Table 6:** Processing of raw tomatoes to produce selected product. Values shown with respect to the Functional Unit.

	Units	Tomato-pasta sauce	Remarks
Functional Unit	kg	1	See Table 1
Inputs			
Raw tomato from wholesales-store	kg	5.65	6% DM
Other raw materials inputs			
Total electricity	kWh	2.66*10^−2^	
Heat consumption, natural gas	MJ	1.88	
Water use	kg	16.04	
Propane	kg	4.3*10^−5^	
Diesel	kg	5.6*10^−5^	
50% Sodium Hydroxide	kg	1.41*10^−4^	[72]
37% Calcium Chloride	kg	2.14*10^−4^	[72]
50% Citric Acid	kg	1.5*10^−7^	[72]
Packaging material			
Bins a	m^3^	3.37*10^−5^	Ingredients transport
Disposable plastic sterile bag	kg	1.29*10^−9^	Ingredients transport
Glass jar	kg	4.24*10^−2^	Consumer pack
PET bottle	kg	8.45*10^−3^	Consumer pack
Metal caps	kg	2.12*10^−3^	Consumer pack
Corrugated tray	kg	9.41*10^−3^	Consumer pack
Composite caps	kg	1.01*10^−3^	Consumer pack
Transport			
Transport-road^b^	t-km	3.22*10^−2^	Transport of ingredients
Transport-rail^b^	t-km	3.76	Transport of ingredients between processors and return of bin
Outputs			
Tomato pasta sauce c	kg	1.3	Total pasta sauce weight = 6.07 kg
Wastewater	m^3^	8.19*10^−3^	
Losses at processing lines (tomato)	kg	5.67*10^−2^	
Packaging waste^d^			
Disposable plastic sterile bag	kg	1.29*10^−9^	
Bins	m^3^	3.37*10^−5^	

Assumptions:

a Bins (@55-gal capacity) assumed for transporting ingredients assumed with 5-year life cycle (reusable).

b Transport of ingredients from processing facility 1 to facility 2 (where ingredients are processed to pasta sauce). Road (plants to warehouse) = 25 km. Rail distance = 2860 km (personal communications).

c Value shown is the tomato portion in the packed tomato pasta sauce. Total pasta sauce weight = 1.3 kg (tomato +ingredients + water). Product yield (tomato portion) = 23% of the raw tomato received at first processor gate (output divided by input at processor).

d Disposal of consumer-based packaging materials was assumed to occur at the consumer level (Appendix 16)

### 2.6 Retail

The LCI for retail (supermarket) primarily accounted for the energy input at supermarkets (Table 7). Annual electricity and natural gas input assumed for supermarkets were 5o kWh per sq.ft and 41 cu. ft per sq. ft [73,74], respectively. Electricity input for food commodities was calculated assuming the following: (i) about 93% of the total electricity was assumed to be related to food sales, covering refrigeration, lighting, ventilation, cooling, heating and operating computers [74] (see Appendix 7.a); (ii) specific energy inputs for each product = energy intensity at supermarket per sq. ft * total supermarket consumer facing area * % of the product specific consumer-facing area [75] divided by total amount of each product sold in the US (see Appendix 7.b). Total number of supermarkets used in the calculation was 15639 (median area of the supermarket is 50009 sq. ft) [73]. The total product sales [76] and supermarket area [73] of the year 2018 were assumed (see Appendix 7.c). Similar steps were followed for calculating the natural gas inputs at the supermarket, but the total energy related to food commodities was considered for heating purpose only (69% of the total natural gas consumed at average U.S supermarkets). Detailed calculations are shown in Appendix 7). Similar approach can also be used to calculate the resource input for fresh products, but considering the sales volume of the respective fresh products from Parr and Daugherty [76].

**Table 7:** LCI for retail. Values shown with respect to the Functional Unit.

		Potato products			
	Units	Potato- chips	Potato-frozen fries	Potato-dehydrated	Tomato product Tomato-pasta sauce
Functional Unit a	kg	1	1	1	1
Inputs					
Product from processor	kg	1.67	1.5	1.3	1.3
Energy inputs^b^					
*Electricity*	kWh	9.3*10^−3^	2.3*10^−2^	1.0*10^−2^	2.7*10^−2^
*Natural gas*		5.4*10^−1^	5.2*10^−1^	6.1*10^−1^	4.41*10^−1^
*Transport* *(processor-retail)-road*	t-km	2	1.8	1.6	1.9
Outputs					
Product^d^	kg	1.56	1.41	1.22	1.22
Biowaste	kg	1.2*10^−1^	1.3*10^−1^	1.2*10^−1^	3.6*10^−1^
Packaging waste					
Corrugated boxes^e^	kg	5.6*10^−2^	3*10^−2^	5.6*10^−2^	9.41*10^−2^

Assumptions:

a See Table 1.

b Energy inputs included electricity consumption for lighting and refrigeration related to the specific products at retail (supermarket) (see Appendix 7). ^c^Road transportation, with the average distance described in Appendix-8. For potato-frozen fries, refrigerated (freezing) transportation in fright lorry was assumed.

d Reference flow, as the final packed product supplied to consumer with respect to FU.

e Corrugated boxes used for the packed products after processing and packaging of final product at processor disposed at the retail stage.

Considering the selected environmental impact categories, the annual refrigerant leakage was ignored for the current estimation, however the rate of leakage can be about 25% of the annual refrigerant loads [77]. It can be calculated considering the typical commercial refrigerant charge in US commercial stores (1588 kg) for a store size of 50009 sq. ft. [77].

### 2.7 Consumer

The consumer transport distance was calculated considering that consumers generally purchase various products in each visit to the retail shop. It is assumed that consumers buy about 30 products per trip, constituting of both food and non-food items. The impact of the transport per product was therefore set at 3.33% of the transport burden [78,79]. At the consumer level, assumptions for estimating the raw materials for preparing the selected products were adapted from [80] (Table 8), which included LCI results for potatoes, but not tomatoes. However, the cooking energy needs depend on the cooking practices and other ingredients that are co-cooked with the main product. The current protocol includes electricity consumption for storage (freezer), cooking/frying and water consumption for washing utensils and plates. Deep frying of frozen fries was accounted with a power output of 2000 W and normal cooking time of 10 minutes (cooking 0.5 kg of product in each batch) consuming 0.67 kWh per kg product. Vegetable oil used (single purpose, without considering further use) was assumed in [80], which was 0.25 kg per kg fries. However, the frying process that we have assumed is on a commercial vendor, which utilizes vegetable oil for multiple frying cycles. In this protocol, after considering the total numbers of hours that a vegetable can be used (i.e. total operating hours of 80 hrs, calculated after daily operation of fryer at 7-8 hrs and running 18 batches per day, with 24% fresh vegetable oil added to the initial volume) the total cycle of fresh vegetable oil (i.e. number of times that vegetable oil is used once filled) was about 4 [81,82]. Hence, the vegetable oil used for frying was 0.06 kg per kg fries for commercial entities preparing fries. In the case of tomato pasta sauce, electricity consumption was assumed as 0.33 kWh per kg (mainly preheating on 2000 W oven) [80]. For water and energy consumption in dishwasher operation it was assumed to be 1.4 kWh per cycle, after further assuming that 20% of the load in each dishwasher cycle is also covered by the cooking utensils and tableware used during the consumption of 1 kg product. Packaging waste was treated in the waste handling model (see Appendix 16).

**Table 8:** LCI for the products use at consumer. Values shown with respect to the Functional Unit.

		Potato products			
	Units	Potato- chips	Potato-frozen fries	Potato-dehydrated	Tomato product Tomato-pasta sauce
Functional Unit	kg	1	1	1	1
Inputs					
Products	kg	1.56	1.41	1.22	1.22
Transport^b^	km	3.33*10^−1^	3.33*10^−1^	3.33*10^−1^	3.33*10^−1^
Preparation^b^					
Electricity for storage, freezer	kWh	-	1.0*10^−2^	-	-
Electricity for cooking/ heating	kWh	-	9.44*10^−1^	-	4.02*10^−2^
Dishwasher, electricity	kWh	3.94*10^−1^	3.94*10^−1^	3.94*10^−1^	3.94*10^−1^
Vegetable oil^b^^,±^	kg	-	0.08	-	-
Water for dishwasher	kg	2.13*10^−2^	2.13*10^−2^	2.13*10^−2^	2.13*10^−2^
Outputs					
Prepared food (FU)	kg	1	1	1	1
Waste					
Bio-waste^c^	kg	5.42*10^−1^	4.7*10^−1^	3.6*10^−1^	1.8*10^−1^
Vegetable oil	kg	-	0.08	-	-
Packaging plastics^d^	kg	2.92*10^−8^	4.1*10^−3^	4.1*10^−3^	-
PET bottle	kg	-	-	-	1.0*10^−2^
Composite caps	kg	-	-	-	1.20*10^−3^
Metal caps/Aluminum portion of MOPP^e^	kg	1.23*10^−15^	-	-	2.12*10^−3^
Glass jar	kg	-	-	-	4.24*10^−2^

**Assumptions:**

^a^Transport distance assumed as (10*3.33%) km (see Appendix 8) (see texts).

b Materials (energy, water) are based on [80]. ^±^ Vegetable oil was accounted after considering the reusability of fresh oil. (cycles for vegetable oil = 4) [81,82] (see texts). Energy inputs were assumed to be the same for each 1 kg processed product.

c Losses at consumer (Appendix 2). For waste handling approach see section 3.

d Packaging materials for the final products packed at processor, disposed at consumer stage.

e Aluminum portion of MOPP disposed at consumer stage.

**Table 9:** LCI for handling biowaste for composting. Biowaste masses, shown in the table represent for the basic scenario. Values shown with respect to the Functional Unit.

		Potato products			
	Units	Potato-chips	Potato-frozen fries	Potato-dehydrated	Tomato product Tomato-pasta sauce
Functional Unit	kg	1	1	1	1
Inputs					
Biowaste total^a^	kg	3.46*10^−1^	3.46*10^−1^	3.46*10^−1^	1.26*10^−1^
Outputs					
Biowaste for compost^b^	kg	1.73*10^−1^	1.73*10^−1^	1.73*10^−1^	6.29*10^−1^
Composting facilities^b^	See footnotes				
Fertilizer values					
N	kg	4.48*10^−4^	4.99*10^−4^	4.56*10^−4^	1.91*10^−3^
P_2_O_5_	kg	1.71*10^−3^	1.9*10^−3^	1.74*10^−3^	7.28*10^−3^
Avoided impacts c					
N-emissions					
*N_2_O*	*kg*	-3.71*10^−5^	-4.13*10^−5^	-3.78*10^−5^	-1.58*10^−4^
*NH_3_*	*kg*	-3.57*10^−5^	-3.98*10^−5^	-3.64*10^−5^	-1.53*10^−4^
*NOx*	*kg*	-2.91*10^−5^	-3.24*10^−5^	-2.96*10^−5^	-1.24*10^−4^
*NO_3_*	*kg*	-6.23*10^−3^	-6.94*10^−3^	-6.35*10^−3^	-2.66*10^−2^

Assumptions:

a Biowaste handling: Biowaste generated at farm to retail assumed for composting.

b Biowaste available for composting = 50% of the total biowaste collected from the supply chain [8]. Infrastructure and materials use in composting facilities based on Ecoinvent v3.6 [8]

c Negative sign indicate the environmental credits to the FU. Avoided impacts due to the substitutions of synthetic fertilizer accounted N emissions following the emission factors reported in [29]. Emissions were calculated as (i) added emissions from compost application (ii) avoided emissions from the equivalent amount of substituted N and P2O5 fertilizers (see Appendix 15, as an example for the estimation steps). Net emissions = added due to compost application plus avoided due to substitution of equivalent N-synthetic fertilizer. P emissions estimation based on LCI guideline [86]. Net emissions are calculated to be zero, since P use efficiency of added compost and substituted synthetic P fertilizer was set to 100% (resulting to net emissions as zero) [39].

## 3 Biowaste handling model and LCIs

Assumptions regarding the waste generated across the supply chain were based on [83], and other sources [59], [60], [61] (Appendix 2). Potatoes and tomatoes were categorized as vegetables. In the integrated supply chain model three alternative biowaste handling scenarios were considered. Features and assumptions for the alternative biowaste management scenarios are shown in Appendix 12. Transportation distance for biowaste to conversion facilities is excluded, considering the high uncertainty on the distances to different conversion facilities in different CRDs.

### 3.1 Waste-to-compost conversion model

It was assumed that 50% of the mass of waste is lost during the composting process [8]. In the base case scenario, the compost model utilizes the waste generated between farm and retail, and waste generated at processor and retail were considered as animal feed. Detail description of the waste handling scenario is presented in Appendix 12.

The compost model was adapted from Ecoinvent v3.6, but the consequences of substituting the equivalent N and P fertilizer in a field (for the added compost and substituted synthetic fertilizer, in terms of N and P emissions) were not considered in the Ecoinvent v3.6 compost model. Since, the use of compost offers environmental credits to the faring system due to substitution of equivalent synthetic fertilizers, we have expanded the system boundary to cover these aspects. The expanded system boundary however also accounted related N and P emissions, following IPCC (2006). To estimate the equivalent fertilizer value, N availability was assumed at 15% [84], whilst 100% was assumed for P fertilizer [85]. Lower N availability in the compost compared to other types of organic fertilizers and synthetic fertilizer, is due to the fact that of the total N concentration minimal amount are in the form of mineral N [50]. NPK content in the compost was adopted from Ecoinvent v3.6 (detailed in Appendix 13).

### 3.2 Waste to biogas and energy conversion model

In the biowaste to energy conversion model, all the biowaste generated in the post-harvest stages of the supply chain is assumed to be collected and transported to a nearby conversion facility (Appendix 8). The farm to retail waste followed composting, as discussed in Section 3.1. Hence, net emissions accounted for both waste management and during the waste to energy conversion. Anaerobic digestion of waste producing biogas was the intermediate product, being further converted to heat and electricity in a combined heat and power plant. LCIs for the energy conversion model are shown in Table 10.

**Table 10:** LCI for handling biowaste for energy conversions. Values shown with respect to the Functional Unit.

		Potato products			
	Units	Potato-chips	Potato-frozen fries	Potato-dehydrated	Tomato product Tomato-pasta sauce
Functional Unit	kg	1	1	1	1
Inputs					
Biowaste total^a^	kg	1.05	1.14	1.05	9.46*10^−1^
Outputs					
Biogas yield^b^	m^3^	1.53*10^−1^	1.79*10^−1^	1.53*10^−1^	5.88*10^−2^
Conversion of biogas c					
Heat input	MJ	9.95*10^−2^	1.16*10^−1^	9.95*10^−2^	3.81*10^−2^
Electricity input	MJ	1.54*10^−2^	1.8*10^−2^	1.54*10^−2^	5.91*10^−3^
Outputs					
Heat	MJ	2.97	3.46	2.97	1.14
Electricity	MJ	1.89	2.2	1.89	7.24*10^−1^
Waste					
Digestate	kg	1.41*10^−1^	1.65*10^−1^	1.41*10^−1^	5.41*10^−2^
Avoided products d					
Crop nutrients (N)	kg	-3.39*10^−4^	-3.95*10^−4^	-3.39*10^−4^	-1.3*10^−4^
Heat and electricity	MJ	as shown in the output above			
Avoided emissions for N-substituted c		See Appendix 15			

Assumptions:

a Biowaste total accounted the total waste generated across the supply chain.

b Biogas yield also accounted the methane losses during anaerobic digestion and from a CHP plants, shown in Appendix 13-14.

c Energy input for the conversion shown in Appendix 13. Fugitive losses (methane leakage) = 1.8% of the total biogas production [87].

d Avoided impacts due to substituting synthetic fertilizer due to application of digestate followed the similar approach, as shown in Table 2. Avoided N = NH4-N* Utilization factor = 2.4 (g NH_4_-N/kg fresh *80%). N Emissions were in the form of (i) added emissions from digestate application (ii) avoided emissions due to the equivalent amount of substituted N fertilizers (with negative sign). See Appendix 15, as an example.

**Table 11:** LCI for handling biowaste for animal feed. Values shown with respect to the Functional Unit.

		Potato products			
	Units	Potato-chips	Potato-frozen fries	Potato-dehydrated	Tomato product Tomato-pasta sauce
Functional Unit^a^	kg	1	1	1	1
Inputs					
Biowaste total	kg	7.08*10^−1^	7.93*10^−1^	7.08*10^−1^	7.06*10^−1^
Outputs					
Animal feed equivalent^c^	kg DM	1.38*10^−1^	1.8*10^−1^	1.38*10^−1^	6.04*10^−3^

Assumptions:

a See Table 1. ^b^Biowaste generated from the processing facility and at retail

c Feed equivalence is selected as corn feed grain. Values are shown in dry matter basis. Detailed assumptions on estimating feed values of the biowaste shown in Appendix 13.

### 3.3 Waste to animal feed conversion model

Since potatoes are a wet feed (with 20% DM), on dry mater basis the equivalent ratio compared to grains is 4.5:1 (i.e., 450 lbs of potatoes equal to 100 lbs of grains) [88]. However, there are different ways of feeding potatoes to cattle, including silage and feeding dried products [89], thus the estimated feed values may vary. Wadhwa et al. [90] argued that because of low fibre content, potatoes instead of forage should be a substitute to grain. As per the general recommendation, 1-2.3 kg of barley or corn is equivalent to 4–5 kg potatoes (i.e. about 18.2-23% of the grain be substituted by potatoes as feed). In the current protocol, after considering the DM content, metabolic energy, protein values and non-digestible fibre, equivalent feed values with respect to corn were calculated (as assumed to be substitutable feed) [91]. Schroeder [91] with an equivalent price of culled potatoes assumed to be an economical substitute for corn, and if fed at moderate level, animal performance is not affected.

Tomatoes are a wet feed (with 6% DM, raw products), the equivalent feed ratio compared to grain was 29%. Bakshi et al. [92] suggested that feeds containing 12.5% waste tomatoes could replace 35% of cereal-based concentrate in diets of lactating goats without affecting apparent nutrient digestibility or product yield. Likewise, they also argued that dried culled tomatoes can replace 3% alfalfa meal in the diets of broilers, and suggested up to 40% tomato pomace (on DM basis) in the diet did not affect the daily weight gain or feed conversion efficiency of the steers [93]. These studies indicate that there are different ways of feeding tomato waste to different livestock, including the conversion of the biomass to silage and alternatively feeding dried products [89,94]. In the current protocol, the feed values were estimated based on the DM content and ME of the waste and the equivalent substitutable conventional feed.

Likewise, in the case of potato supply chain, the starch generated from the processing lines was also handled in the waste handling component of the integrated model (Table 12). Although corn starch is less expensive than starch available from other sources, because of unique properties of potato starch, it can be regarded as potential alternative in certain applications [95]. Stearns et al. [95] also argued that if potato starch becomes available at corn starch prices, it would be preferred in most applications.

**Table 12:** LCI for handling of starch generated from potato processing lines. Values shown with respect to the Functional Unit.

		Potato products		
	Units	Potato-chips	Potato-frozen fries	Potato-dehydrated
Functional Unit	kg	1	1	1
Inputs				
Starch from processor^a^	kg	3.33*10^−2^	2.58*10^−1^	2.57*10^−1^
Outputs				
Equivalent starch available in the market^b^	kg	2.52*10^−3^	2.0*10^−2^	1.99*10^−2^

Assumptions:

a See Table 5.

b Equivalent starch values were estimated considering the DM adjustments for maize based starch (86%) [8] and potato-recovered starch from the processing line (6.65% DM) (Appendix 10).

## 4 Synchronization and operation of integrated model

All the three major components of the integrated model with their related LCIs were handled in macro-enabled MS-excel program. A separate programming code was developed to connect all the LCA model components and transferring the data to the chosen LCA tool (SimaPRO-9).

## 5 Evaluation of future scenarios

Environmental LCAs of the selected F&V supply chain are evaluated for the current and the future climatic scenarios. The scenarios included crop modeling results, such as median, 25^th^ and 75^th^ percentile values of the crop yields and irrigation water requirement (for the years 2017, 2030 and 2050). A detail description of the climatic scenarios can be found in Crop Model [7]. In the future scenarios, it is anticipated that higher atmospheric CO_2_ concentration will enhance crop growth, provided that the crop nutrient supply is not limited. In the crop model, the annual atmospheric CO_2_ concentration changes were projected for the baseline (from 1981 to 2010) and for the future scenarios (years 2030 and 2050). Future projections were made under the RCP8.5 scenario, utilizing five GCM's models Gustafson et al. [14]. Crop yield projection included the integration of crop models with the economic model, also following the effects of technological advancement and of the adaptation measures (mainly altering the cropping calendar) on the crop yields; further elaborated in Gustafson et al. [14]. The crop yields data for the future scenarios is shown in Appendix 1.b.

## Declaration of Competing Interest

The authors declare that they have no known competing financial interests or personal relationships which have or could be perceived to have influenced the work reported in this article.

## Appendix Supplementary materials

Image, application 1
