# Corrigendum to “protocol for life cycle assessment modeling of US fruit and vegetable supply chains- cases of processed potato and tomato products” [Data in Brief 34 (2021) 1-24/ 106639]

**DOI:** 10.1016/j.dib.2021.107223

**Published:** 2021-06-12

**Authors:** Ranjan Parajuli, Dave Gustafson, Senthold Asseng, Claudio O. Stöckle, John Kruse, Chuang Zhao, Pon Intrapapong, Marty D Matlock, Greg Thoma

**Affiliations:** aRalph E. Martin Department of Chemical Engineering, University of Arkansas, Fayetteville, AR 72701, USA; bAgriculture & Food Systems Institute, Washington, DC 20005, USA; cTechnical University Munich, World Agricultural Systems Center, 85354 Freising, Germany; dDepartment of Biological Systems Engineering, Washington State University, Pullman, WA 99164-6120, USA; eWorld Agricultural Economic and Environmental Services, LLC, 3215 S. Providence Rd, Suite 3 Columbia, MO 65203, USA; fAgricultural and Biological Engineering Department, University of Florida, Gainesville, FL 32611, USA; gDepartment of Biological and Agricultural Engineering, University of Arkansas, Fayetteville, AR 72701, USA

The authors regret that while presenting the data in the Supplementary information (Parajuli R et al. 2021a), there were some errors (in terms of units and the corresponding values). We also realized to provide some additional clarities to the production processes. Following amendments and description on the inputs are mainly covered in this Corrigendum.1.Caption for the Fig 3 (in Parajuli R et al. 2021a) is edited as “Fig 3. Mass flow balance for the fryer. Mass of materials (*ṁ_n_)* are shown in Table A-3. Method based on (Wu et al., 2010). Mass flow rate (kg per second) of the raw materials in the fryer: *ṁ_1_ = oil input, ṁ_2_* = oil return, *ṁ_3_ = fines removal, ṁ_4_* = air inflow, *ṁ_5_* = frying vapors, *ṁ_6_* = raw potato input, *ṁ_7_* = fried potatoes output. Masses are shown in Appendices 9-10.”2.Caption for Appendix-9 is edited as “Appendix-9. Processing of the potato to produce potato-chips. Method for calculating the energy input as per the mass flow rates inside the fryer based on (Wu et al., 2010)”.3.In Table 7 (in Parajuli R et al. 2021a), “unit for the consumption of natural gas” should be written as “Mega Joule (MJ)”. Accordingly, unit for “natural gas” (mentioned in Appendix 7.a) should be in “MJ/sq. ft”.4.Energy inputs in (Table 5, in Parajuli R et al. 2021a): (i) electricity input per functional unit, respectively for potato-chips, frozen fries and dehydrated flakes should be 1.42 kWh, 0.63 kWh and 0.002 kWh, and (ii) heat (natural gas) input per functional unit, respectively for potato-chips, frozen fries and dehydrated flakes should be used as 1.31 MJ, 1.3 MJ and 1.3 MJ. Total energy input: potato-chips (6.42 MJ), frozen fries (3.55 MJ) and dehydrated flakes (1.31 MJ). To understand the influence of raw materials used on the calculated environmental impacts, e.g., the “sensitivity of input parameters to GHG emissions” including the energy input at processor were originally discussed in Parajuli R et al. 2021 (b). This might help to estimate the footprints of the selected products for the varying raw materials inputs, including energy.5.For clarity to the production process:a.it should be noted that potato-fries are first par-fried at the processor stage (detailed in Parajuli R et al. 2021a), which are again deep-fried in vegetable oil at consumer stage. Multiple batch frying was accounted with estimated refilling rate of vegetable oil at commercial vendors (detailed in Parajuli R et al. 2021a).b.the production process at the processor assumed the simultaneous operation of three processing lines, which thus followed the “subdivisions of the resource use” among the products.c.In the Appendix 9 (Parajuli R et al. 2021a), for the calculation of energy inputs (for chips), temperature of frying oil (T_fo_) was assumed as 150 °C. Temperature of raw materials (T_6_) was assumed at 57 °C. Energy input for processing the frozen fries were accounted based on the mass and energy balance estimates, as suggested in Fooddelphi (2018).

The authors would like to apologise for any inconvenience caused. We are thankful to the received suggestions to enhance the clarity to the published data.
