# Supplementary material for: Protocol for life cycle assessment modeling of US fruit and vegetable supply chains- cases of processed potato and tomato products
Source: Data Brief. 2020 Dec 10;34:106639. doi: 10.1016/j.dib.2020.106639 (PMC7749376; doi:10.1016/j.dib.2020.106639)
Supplement: Supplementary file 1 [file mmc1.docx]

**Supplementary information-**Data used for computing the reference flows in the processed potato and tomato products supply chain.

Protocol for Life Cycle Assessment Modeling of US Fruit and Vegetable Supply Chains- cases of processed potato and tomato products

**Authors**:

Ranjan Parajuli^1*^, Dave Gustafson^2^, Senthold Asseng^3^, Claudio O. Stöckle ^4^, John Kruse^5^, Chuang Zhao^3^, Pon Intrapapong^5^, Marty D Matlock^6^, Greg Thoma^1^

**Affiliations**:

*^1^Ralph E. Martin Department of Chemical Engineering, University of Arkansas, Fayetteville, AR 72701, USA*

*^2^Agriculture & Food Systems Institute, Washington, DC, 20005, USA*.

*^3^Agricultural and Biological Engineering Department, University of Florida, Gainesville, Florida 32611, USA,*

*^4^Department of Biological Systems Engineering, Washington State University, Pullman, WA 99164-6120, USA*

*^5^World Agricultural Economic and Environmental Services, LLC, 3215 S. Providence Rd, Suite 3 Columbia, MO 65203*

*^6^Department of Biological and Agricultural Engineering, University of Arkansas, Fayetteville, AR 72701, USA*

**Contact email**: *rparajul@uark.edu*

**Appendix 1.** Selected Crop Reporting Districts (CRDs) for processing potato and tomato

Appendix 1.a. On-farm characteristics of the selected CRDs. Data computed based on Crop model for the baseline scenario (year 2017). Blank space in the table are intentional. Regions included in the crop modeling are the CRDs, where the major commercial production is occurring.

| State | CRDs | Processing-Potatoes | | | | |  | Processing-Tomatoes | | | | |
| --- | --- | --- | --- | --- | --- | --- | --- | --- | --- | --- | --- | --- |
|  |  | Yield (tons/ha) | N-inputs (kg/ha) | P2O5 inputs  (kg per ha) | K2O inputs  (kg per ha) | Irrigation (m3/ha) |  | Yield (tons/ha) | N-inputs (kg/ha) | P2O5 inputs (kg per ha) | K2O inputs (kg per ha) | Irrigation (m3/ha) |
| Arizona | AZ80 | 32.67 | 217.32 | 27.71 | 225.85 | 3074 |  | - | - | - | - | 0 |
|  |  |  |  |  |  |  |  |  |  |  |  |  |
| California | CA40 | - | - | - | - | - |  | 113.04 | 165.87 | 279.67 | 251.70 | 2735 |
|  | CA50 | - | - | - | - | - |  | 103.22 | 151.45 | 251.41 | 226.27 | 3687 |
|  | CA51 | 54.63 | 363.39 | 46.33 | 377.58 | 5930 |  | 114.00 | 167.27 | 289.74 | 260.77 | 4329 |
|  | CA80 | 36.66 | 243.83 | 31.09 | 267.79 | 3482 |  | 111.14 | 163.08 | 272.33 | 245.09 | 4171 |
|  |  |  |  |  |  |  |  |  |  |  |  |  |
| Colorado | CO80 | - | - | - | - | - |  | - | - | - | - | - |
|  |  |  |  |  |  |  |  |  |  |  |  |  |
| Florida | FL50 | 27.2 | 180.96 | 23.07 | 200.31 | 625 |  | 110.48 | 162.11 | 282.84 | 254.56 | 3204 |
|  | FL80 | 29.14 | 193.85 | 24.71 | 189.13 | 995 |  | 110.48 | 162.11 | 291.46 | 262.31 | 3150 |
|  |  |  |  |  |  |  |  |  |  |  |  |  |
| Georgia | GA70 | - | - | - | - | - |  | - | - | - | - | - |
|  |  |  |  |  |  |  |  |  |  |  |  |  |
| Idaho | ID70 | 61.75 | 410.79 | 52.37 | 422.77 | 6398 |  | - | - | - | - | - |
|  | ID80 | 52.02 | 346.04 | 44.12 | 344.21 | 5282 |  | - | - | - | - | - |
|  | ID90 | 46.01 | 306.08 | 39.02 | 337.59 | 4231 |  | - | - | - | - | - |
|  |  |  |  |  |  |  |  |  |  |  |  |  |
| Maine | ME10 | 34.85 | 231.80 | 29.55 | 253.62 | 376 |  | - | - | - | - | - |
|  |  |  |  |  |  |  |  |  |  |  |  |  |
| Michigan | MI50 | 43.63 | 290.20 | 37.00 | 301.05 | 1698 |  | 78.21 | 114.77 | 158.93 | 143.04 | 1860 |
|  | MI80 | 50.8 | 337.90 | 43.08 | 387.52 | 2127 |  | 77.22 | 113.31 | 154.80 | 139.32 | 1658 |
|  |  |  |  |  |  |  |  |  |  |  |  |  |
| Minnesota | MN40 | 51.3 | 341.27 | 43.51 | 375.51 | 1674 |  | - | - | - | - | - |
|  | MN50 | 48.18 | 320.46 | 40.86 | 296.83 | 1354 |  | - | - | - | - | - |
|  | MN80 | 31.06 | 206.63 | 26.34 | 213.90 | - |  | - | - | - | - | - |
|  | MN90 | 50.74 | 337.53 | 43.03 | 346.21 | 2277 |  | - | - | - | - | - |
|  |  |  |  |  |  |  |  |  |  |  |  |  |
| New York | NY40 | 32.4 | 215.55 | 27.48 | 207.00 | 313 |  | 59.50 | 87.31 | 146.76 | 132.09 | 917 |
|  |  |  |  |  |  |  |  |  |  |  |  |  |
| North Dakota | ND30 | 31.03 | 206.43 | 26.32 | 224.62 | 648 |  | - | - | - | - | - |
|  |  |  |  |  |  |  |  |  |  |  |  |  |
| Oregon | OR10 | 43.67 | 290.50 | 37.04 | 307.17 | 2430 |  | 110.86 | 162.67 | 294.17 | 264.76 | 3391 |
|  | OR30 | 71.53 | 475.80 | 60.66 | 491.83 | 6848 |  | - | - | - | - | - |
|  |  |  |  |  |  |  |  |  |  |  |  |  |
| Texas | TX97 | 45.62 | 303.45 | 38.69 | 370.23 | 3579 |  | - | - | - | - | - |
|  |  |  |  |  |  |  |  |  |  |  |  |  |
| Washington | WA10 | 43.88 | 291.88 | 37.21 | 335.51 | 1812 |  | 110.48 | 162.11 | 337.31 | 303.58 | 3207 |
|  | WA20 | 74.64 | 496.50 | 63.30 | 498.77 | 7246 |  | 110.48 | 162.11 | 286.64 | 257.97 | 3861 |
|  | WA50 | 71.04 | 472.56 | 60.25 | 462.98 | 6905 |  | 103.47 | 151.82 | 265.15 | 238.64 | - |
|  | WA90 | 90.03 | 598.89 | 76.36 | 659.16 | 8636 |  | - | - | - | - | - |
|  |  |  |  |  |  |  |  |  |  |  |  |  |
| Wisconsin | WI30 | 41.94 | 278.97 | 35.57 | 290.61 | 646 |  | - | - | - | - | - |
|  | WI50 | 55.31 | 367.94 | 46.91 | 385.47 | 1780 |  | - | - | - | - | - |
|  | WI60 | - | - | - | - | - |  | - | - | - | - | - |

Appendix 1.b. Yield and irrigation water, current and projected future scenarios for the selected CRDs. Data computed based on Crop model. Blank space are the regions excluded in the crop modeling, where the major commercial production is not occurring.

| CRDs | Counties | 2017 | | | | | 2030 | | | | | 2050 | | | |
| --- | --- | --- | --- | --- | --- | --- | --- | --- | --- | --- | --- | --- | --- | --- | --- |
|  |  | Potato | | | Tomato | | Potato | | Tomato | | | Potato | | Tomato | |
|  |  | Yield  (t/ha) | Irrigation  (m3/ha) | Yield  (t/ha) | | Irrigation  (m3/ha) | Yield  (t/ha) | Irrigation  (m3/ha) | Yield  (t/ha) | Irrigation  (m3/ha) | Yield  (t/ha) | | Irrigation  (m3/ha) | Yield  (t/ha) | Irrigation  (m3/ha) |
| AZ80 | Maricopa | 32.67 | 3074.10 | - | | - | 34.55 | 2634.33 | - | - | 37.64 | | 2550.70 | - | - |
| CA40 | Fresno | - | - | 113.04 | | 2734.98 | - | - | 128.04 | 2805.28 | - | | - | 139.84 | 2976.99 |
| CA50 | Imperial | 0.00 | - | 103.22 | | 3687.27 | - | - | 116.62 | 3578.20 | - | | - | 125.71 | 3541.27 |
| CA51 | Monterey | 54.63 | 5930.72 | 114.00 | | 4328.88 | 58.58 | 4728.57 | 132.78 | 4298.63 | 62.93 | | 4264.02 | 144.88 | 4274.21 |
| CA80 | Yolo | 36.66 | 3481.69 | 111.14 | | 4170.60 | 40.27 | 3122.22 | 123.93 | 3909.31 | 44.64 | | 3171.79 | 136.17 | 3917.85 |
| FL50 | - | 27.20 | 625.52 | 110.48 | | 3204.48 | 30.10 | 636.55 | 128.46 | 3260.17 | 33.39 | | 774.34 | 141.43 | 3343.71 |
| FL80 | - | 29.14 | 995.09 | 110.48 | | 3150.24 | 30.07 | 795.86 | 130.24 | 3281.19 | 31.52 | | 803.19 | 145.74 | 3477.16 |
| ID70 | Bingham | 61.75 | 6398.38 | - | | - | 66.05 | 6263.53 | - | - | 70.47 | | 6142.27 | - | - |
| ID80 | Canyon | 52.02 | 5281.94 | - | | - | 55.59 | 5169.22 | - | - | 57.37 | | 4981.96 | - | - |
| ID90 | Minidoka | 46.01 | 4230.90 | - | | - | 52.06 | 4504.60 | - | - | 56.27 | | 4576.02 | - | - |
| ME10 | Aroostook | 34.85 | 376.08 | - | | - | 37.81 | 483.67 | - | - | 42.27 | | 909.98 | - | - |
| MI50 | Montcalm | 43.63 | 1698.32 | 78.21 | | 1859.54 | 46.96 | 1832.52 | 78.43 | 1642.01 | 50.18 | | 2101.14 | 79.47 | 1609.62 |
| MI80 | St. Joseph | 50.80 | 2127.42 | 77.22 | | 1657.54 | 58.85 | 2748.06 | 77.03 | 1597.42 | 64.59 | | 3242.76 | 77.41 | 1569.47 |
| MN40 | Dakota | 51.30 | 1674.41 | - | | - | 57.89 | 2024.45 | - | - | 62.59 | | 2353.11 | - | - |
| MN50 | Freeborn | 48.18 | 1354.31 | - | | - | 49.99 | 1561.50 | - | - | 49.48 | | 1506.24 | - | - |
| MN80 | Otter Tail | 31.06 | - | - | | - | 33.83 | 0.00 | - | - | 35.65 | | 23.64 | - | - |
| MN90 | Renville | 50.74 | 2277.55 | - | | - | 54.92 | 2492.83 | - | - | 57.71 | | 2594.49 | - | - |
| NY40 | Genesee | 32.40 | 313.46 | 59.50 | | 916.76 | 33.28 | 200.95 | 67.55 | 1085.72 | 34.50 | | 297.93 | 73.39 | 1256.67 |
| ND30 | Walsh | 31.03 | 648.71 | - | | - | 35.09 | 723.58 | - | - | 37.44 | | 867.66 | - | - |
| OR10 | Marion | 43.67 | 2430.82 | 110.86 | | 3391.42 | 47.71 | 2191.74 | 131.98 | 3525.37 | 51.20 | | 2022.10 | 147.10 | 3752.71 |
| OR30 | Umatilla | 71.53 | 6848.82 | - | | - | 78.37 | 6883.88 | - | - | 81.98 | | 6747.56 | - | - |
| TX97 | Hidalgo | 45.62 | 3579.22 | - | | - | 55.01 | 3743.61 | - | - | 61.71 | | 3966.77 | - | - |
| WA 10 | Benton | 43.88 | 1812.34 | 110.48 | | 3206.51 | 50.69 | 1960.81 | 140.28 | 3321.28 | 55.92 | | 1866.10 | 168.67 | 4000.93 |
| WA20 | Grant | 74.64 | 7246.14 | 110.48 | | 3860.55 | 79.25 | 7152.07 | 129.08 | 4146.20 | 83.13 | | 7047.64 | 143.33 | 4298.56 |
| WA50 | Skagit | 71.04 | 6904.74 | 103.47 | | - | 73.81 | 6643.04 | 120.48 | - | 77.17 | | 6450.67 | 132.59 | 0.00 |
| WA90 | Walla Walla | 90.03 | 8636.26 | - | | - | 100.19 | 8874.64 | - | - | 109.87 | | 9026.40 | - | - |
| WI30 | Fond du Lac | 41.94 | 645.87 | - | | - | 45.36 | 860.82 | - | - | 48.44 | | 1076.28 | - | - |
| WI50 | Langlade | 55.31 | 1780.01 | - | | - | 60.44 | 2083.12 | - | - | 64.25 | | 2401.73 | - | - |

Appendix 1.c. Assumptions made on the breakdown of NPK fertilizer inputs (by types). Data computed based on Wernet et al. (2016).

| Fertilizer types | Potato | Tomato |
| --- | --- | --- |
| N-fertilizer |  |  |
| Ammonia, liquid | 39% | 7.7% |
| Urea, as N | 14% | 28% |
| Ammonium nitrate, as N | 19% | 0.5% |
| Nitrogen fertiliser, as N | 28% | 40.4% |
| Ammonium sulfate | - | 23.4% |
| P-fertilizer |  |  |
| Phosphate fertiliser, as P2O5 | 100% | 99% |
| Phosphate rock | - | 1% |
| K-fertilizer |  |  |
| Potassium chloride, as K2O | 100% | 59.1% |
| Potassium fertiliser, as K2O | - | 27.7% |
| Potassium sulfate, as K2O | - | 13.3% |

Appendix 1.d. Assumptions made on the breakdown of pesticides inputs for potato production. Data computed based on Wernet et al. (2016) and Crop Budget Reports.

| Pesticides class/active ingredients | % of the total inputs |
| --- | --- |
| Pesticide, unspecified | 56.31% |
| Acetamide | 0.28% |
| Bipyridylium-compound | 0.18% |
| Dithiocarbamate-compound | 3.42% |
| Dinitroaniline-compound | 0.26% |
| Glyphosate | 0.01% |
| Organophosphorus compound | 1.65% |
| Pyridazine-compound | 0.23% |
| Phenoxy-compound | 0.13% |
| [sulfonyl]urea-compound | 0.05% |
| [thio]carbamate-compound | 37.11% |
| Triazine-compound, unspecified | 0.37% |

Appendix 1.e. Assumptions made on the breakdown of pesticides inputs for tomato production. Data computed based on Wernet et al. (2016) and Crop Budget Reports.

| Pesticides class/active ingredients | % of the total inputs |
| --- | --- |
| Trifluralin | 10% |
| Lambda cynalothirin | 7% |
| Glyphosate | 46% |
| Chlorothalonil | 15% |
| Rimsulfuron | 1% |
| Oxyfluorfen | 1% |
| Metolachlor | 10% |
| Copper Hydroxide | 9% |
| Adjuvant | 1% |

**Appendix 2.** Losses at different stages of supply chain (Buzby et al., 2011; Buzby et al., 2009; Buzby and Hyman, 2012; USDA, 2019)

| F&V products | Loss (%) |
| --- | --- |
| Farm-retail loss |  |
| **Vegetables^1^** | 18% |
| Retail loss |  |
| **Vegetables** |  |
| *Fresh* | *10* |
| *Processed* | *6%* |
| Consumer loss: |  |
| **Vegetables** |  |
| *Fresh* | *24%* |
| *Processed* | *18%* |
| Wholesales/storage loss (shrinkages) |  |
| Potatoes and tomatoes (Harvey, 1978) | 2.07% (0-4.9%) |

**^1^ Description on the food loss**:

Losses occurring at the post-harvest stages of the supply chain (retail and consumer) for the fresh (24%) and processed categories (18%) were adopted from the studies (Buzby et al., 2011; Buzby et al., 2009; Buzby and Hyman, 2012). In most of the studies dealing with the food loss/waste management, often the post-harvest stages are only accounted, despite there is consensus on the fact that on-farm losses are significant (Minor et al., 2020). The estimated loss of F&V during the agricultural production and harvest in the N. America was reported to be 30% (Minor et al., 2020). In the same study, the rest of the supply chain (retail and consumer) followed the similar shares, as shown in the appendix 2. Furthermore, when various fresh and processed (mainly canned, frozen and dehydrated products) were evaluated, the losses occurring between farm and retail ranged from 9 % (for fresh) and 27% (for processed varieties, which averaged at 18%). This estimation was based on the data “Loss-Adjusted Food Availability Documentation” (<https://www.ers.usda.gov/data-products/food-availability-per-capita-data-system/>) for the both fresh and processed categories. Elik et al. (2019) also reported that losses due to harvesting for fruits and vegetables (in general) was 20%. So, we choose 18% to represent the losses for vegetables (between farm and retail).

**Appendix 3. Conveyance efficiency of different irrigation technologies**

| Technologies | Efficiency |
| --- | --- |
| Central pivot | 85% |
| Furrow (conventional) | 55% |
| Drip |  |
| Sub-surface | 95% |
| Surface | 90% |
| Micro spray | 90% |

**Appendix 4.** Assumptions on resource use for installing drip irrigation system, values shown per m^3^ water applied.

|  | Unit | Amount |
| --- | --- | --- |
| Water application |  |  |
| Irrigation water application | m^3^ | 1.00 |
| Water pumping | MJ/m^3^ | 8.50*10^-1^ |
| Chemicals and fertilizer |  |  |
| Sulfuric acid | kg/m^3^ | 8.26*10^-2^ |
| Acid pumping | MJ/m^3^ | 8.55*10^-3^ |
| Sodium hypochlorite (bleach) | kg/m^3^ | 1.09*10^-3^ |
| Bleach pumping | MJ/m^3^ | 3.72*10^-4^ |
| Fertilizer pumping | MJ/m^3^ | 3.71*10^-5^ |
| Materials for infrastructure |  |  |
| Polyethylene ^a^ | kg/m^3^ | 1.58*10^-3^ |
| Polyvinyl chloride ^a^ | kg/m^3^ | 1.44*10^-4^ |
| **Assumptions:**  Calculations based on Refs., including (Eranki et al., 2017; Holmer and Schnitzler, 1997; Maughn et al., 2017; Mitchell et al., 2014; Munoz-Carpena et al., 2003; Schwankl et al., 1991; Simonne et al., 2008). Life time of the drip tapes and pipe were assumed as 5 years for polyethylene and 25 years for PVC (https://www.agrifutures.com.au/wp-content/uploads/publications/14-046.pdf)  Other sources:  <https://extension.psu.edu/determining-how-long-to-run-drip-irrigation-systems-for-vegetables>  http://smartirrigation.co.nz/wp-content/uploads/2014/03/INZ-Bk7-DripIrrigation-Online.pdf | | |

**Appendix 5.** Assumptions for the estimation of the packaging materials

Appendix 5.1. Dimensions of the corrugated box for handling raw products from farm to proccessor.

|  | Units | Specifications | References |
| --- | --- | --- | --- |
| **Cartoon** |  |  | (Vzw, 2014) (ASTM Standards, 2018) |
| Dimension | cm | 38 x 26 x 21 | L*W*H  <https://www.uline.com/Product/Detail/S-20444/Food-Containers/Tomato-Boxes-25-lb> |
| Weight of the material | kg | 0.4 |  |
| Buk density | kg m^-3^ | 60 | <https://www.rcbc.ca/files/u3/aa_facts-cardboard.pdf>  <https://www.aqua-calc.com/page/density-table/substance/cardboard>\ |
| **Wooden pallets** |  |  |  |
| Dimensions | cm | 120 x 120 | L x W:  (ASTM Standards, 2018); <https://www.freightquote.com/how-to-ship-freight/standard-pallet-sizes>  Density of pallets: 550 kg per m^3^ (Vzw, 2014). |
| Capacity to handle product | kg | 2087 | (Vzw, 2014) |
| Weight of the pallets as per the handling capacity | kg | 18 |  |

Appendix 5.2. Assumptions for packing 1o00 grams of chips

| Materials^a^ | Units | Amount |
| --- | --- | --- |
| OPP^b^ | kg per kg product | 1.77*10^-8^ |
| MOPP^b^ | kg per kg product | 1.15*10^-8^ |
| Aluminum | kg per kg product | 1.23*10^-15^ |
| Corrugated box^b^ | kg per kg product | 5.6*10^-2^ |
| Assumptions:  ^a^ Calculation based on the parameters shown in Table 5.2.1. Calculations based on Refs. (Carvalho et al., 2018; Nilsson et al., 2011)  ^b^ Both OPP and MOPP considered under the category Packaging plastic film (LDPE).  ^c^ Corrugated box, assumed as shown in Table 5.5. | | |

Appendix 5.2.1. Data for packaging 200-gram chips pack

| Materials^a^ | Units | Amount | Remarks |
| --- | --- | --- | --- |
| Outer layer |  |  |  |
| OPP (oriented polypropylene)^b^ | m | 3*10^-5^ | 30 µm thick |
| Mass of OPP | kg | 3.55*10^-9^ | Volume *Density |
| Density | kg/ m^3^ | 9.1*10^-4^ |  |
| Inner layer |  |  |  |
| Metallized oriented polypropylene (metallized OPP)^b^ | m | 2.3*10^-9^ | 28 µm thick |
| Density of plastic | kg/ m^3^ | 6.3*10^-4^ |  |
| Mass of MOPP | kg | 2.29*10^-9^ |  |
| Aluminum^c^ | m | 7.0*10^-7^ | 0.7 µm thick |
| Surface area of 200 g crisp packet | m^2^ | 0.13 | m2 |
| Volume of aluminum | m^3^ | 9.1*10^-8^ | m^3^ |
| Density of Aluminum | kg/ m^3^ | 2.71*10^-9^ |  |
| kg of aluminum | kg | 2.47*10^-16^ |  |
| **Assumptions:**  ^a^ Data based on: <http://www.diva-portal.org/smash/get/diva2:702819/FULLTEXT01.pdf>  ^b^ Both OPP and MOPP were modelled as Packaging plastic film (LDPE) from Ecoinvent.  ^c^ Mass of aluminum used in the MOPP is also modelled considering the aluminum content. Energy used for gluing it to the plastic is not considered, due to unavailability of any data on this. | | | |

Appendix 5.3. Assumptions for packing 1 kg potato frozen fries

| Materials | Unit | Amount |
| --- | --- | --- |
| Polyethylene bag^a^ | kg per kg product | 0.0041 |
| Corrugated box^a^ | kg per kg product | 3*10^-2^ |
| **Assumptions:**  ^a^ Data based on: (Mouron et al., 2016) . | | |

Appendix 5.4. Assumptions for packing 1 kg potato dehydrated

| Materials | Units | Amount |
| --- | --- | --- |
| Plastic bag (capacity of hold 1 kg potato)^a^ | kg | 1.77*10^-8^ |
| Corrugated box per kg product ^b^ | kg | 5.6*10^-2^ |
| **Assumptions:**  ^a^ Pouch sizing, assumed as OPP considered for potato-chips (see Appendix 4.2)  ^b^ Corrugated box, assumed as shown in Table c. | | |

Appendix 5.5. Assumptions for the corrugate box used at processor

| Specification | Units | Amount |
| --- | --- | --- |
| Total weight handling capacity of the box ^a^ | kg | 22.4 |
| Volume of box | m^3^ | 0.21 |
| Density of box | kg/m^3^ | 60 |
| Total weight | kg | 1.26 |
| Corrugated box handling capacity^b^ | kg | 22.5 |
| **Assumptions:**  ^a^ Capacity of box : 14-25 kg <https://www.samsclub.com/p/valley-select-crinkle-cut-french-fries-6-5-lb/134062>  Properties of the box:  <https://www.rcbc.ca/files/u3/aa_facts-cardboard.pdf>  <https://www.aqua-calc.com/page/density-table/substance/cardboard>  <https://en.wikipedia.org/wiki/Corrugated_fiberboard> | | |

**Appendix 6.** Cooling load calculations at warehouse storage.

6-a. Calculation of cooling load at storage, based on (Kenneth et al., 2016; Kraemer et al., 2015; Krishnakumar T., 2002; Voss et al., 2001). For calculation purpose 100 tons of raw products were considered (refrigeration capacity per 1 kg raw potato shown at the bottom).

| Parameters | Units | Potatoes | Tomatoes* |
| --- | --- | --- | --- |
| Optimum storage temperature | °C | 7.25 | 15.5 |
| Optimal relative humidity (%) | % |  |  |
| Approximate cold storage, duration | days |  |  |
| Quantity to be stored | tons | 100 | 100 |
| Daily loading rate | tons | 20 | 20 |
| Ambient conditions |  |  |  |
| Temperature | *°C* | 30.35 | 30.35 |
| Properties of the F&V products |  |  |  |
| Bulk density | kgm^-3^ | 641 | 481 |
| Heat of respiration | kCal/ton/24 h | 851 | 1484 |
| Specific heat (20%M.C) | kCal/kg°C | 0.84 | 0.95 |
| **Design of Cold Storage** |  |  |  |
| Assumed inner dimension of storage facility |  |  |  |
| Length | m | 18 | 14 |
| Breadth | m | 15 | 8 |
| Height | m | 10 | 5 |
| Total internal volume | m^3^ | 2700 | 560 |
| Free Volume available inside the storage | m^3^ | 2451 | 259 |
| Assumed external dimension of storage facility |  |  |  |
| thickness of wall | m | 0.5 | 0.5 |
| Length | m | 19 | 15 |
| Breadth | m | 16 | 9 |
| Height | m | 10.7 | 5.7 |
| Total external volume | m^3^ | 3253 | 770 |
| Total building volume | m^3^ | 801 | 510 |
| **Heat transfer through buildings** |  |  |  |
| **Heat transfer through wall** |  |  |  |
| Surface area | m^2^-length | 360 | 140 |
| Surface area | m^2^-width | 570 | 240 |
| Total Surface area | m^2^ | 930 | 380 |
| Insulation resistance to heat | R | 16 | 16 |
| Thickness of brick | m | 0.44 | 0.44 |
| Thermal conductivity of the brick | kCal/m/h ^o^C | 0.62 | 0.62 |
| Thickness of the cement plaster | m | 0.01 | 0.01 |
| Thermal conductivity of the cement plaster | kCal/m/h ^o^C | 1.49 | 1.488 |
| Overall heat transfer coefficient (U) | kCal/m^2^/h ^o^C | 1.39 | 1.39 |
| Heat transfer through building material (Q) | kCal/24 hr | 719700 | 189046 |
| **Heat transfer through insulation material** |  |  |  |
| Q, through insulating materials | kCal/24h | 32224 | 8465 |
| Total heat transfer through walls | kCal/24h | 751925 | 197510 |
| Total heat transfer through walls | kCal/16h | 501283 | 131674 |
| Total heat transfer through walls | kCal/h | 31330 | 8230 |
| **Heat transfer through ceiling** |  |  |  |
| Surface area | m^2^ | 270 | 112 |
| Insulation resistance to heat | R | 20 | 20 |
| Thickness of the cement concrete | m | 0.2 | 0.2 |
| Thermal conductivity of the cement concrete | kCal/m/h ^o^C | 1.49 | 1.49 |
| Thermal adjustment for ceiling |  | 0.2 | 0.2 |
| heat transfer through ceiling material | kCal/24h | 250734 | 66863 |
| Heat transfer through insulation material | kCal / 24 | 7484 | 1996 |
| Total heat transfer through ceiling | kCal/24h | 258218 | 68858 |
| Total heat transfer through ceiling | kCal/16h | 172146 | 45906 |
| Total heat transfer through ceiling | kCal/h | 1*10^4^ | 3*10^3^ |
| **Heat transfer through Floor** |  |  |  |
| Surface area | m^2^ | 3*10^2^ | 1*10^2^ |
| Insulation resistance to heat | R | 1*10^1^ | 1*10^1^ |
| Thickness of the cement concrete | m^2^ | 2 | 2 |
| Thermal conductivity of the cement concrete | kcal/m/h ^o^C | 1 | 1 |
| heat transfer through floor material | kCal/24h | 1*10^4^ | 4*10^3^ |
| Total heat transfer through floor | kCal/16h | 9*10^3^ | 2*10^3^ |
| Total heat transfer through floor |  | 6*10^2^ | 2*10^2^ |
|  |  |  |  |
| Total heat transfer through floor+ceiling+walls | kCal/24h | 1*10^6^ | 3*10^5^ |
| Total heat transfer through walls | kCal/16h | 7*10^5^ | 2*10^5^ |
| Total heat transfer through floor+ceiling+walls | kCal/h | 4*10^4^ | 1*10^4^ |
| **Product Load** |  |  |  |
| Product cooling | kCal/24 h | 4*10^5^ | 3*10^5^ |
| Total product load | kCal/16 h | 3*10^5^ | 2*10^5^ |
| Total product load | kCal/h | 3*10^4^ | 2*10^4^ |
| **Respiration Load** |  |  |  |
| Average temperature | ^o^C | 18.8 | 22.93 |
| Rate of heat of respiration | kCal /ton/24 h | 8.51*10^2^ | 1.48*10^3^ |
| Respiratory heat load | kCal /24 h | 8.51*10^4^ | 1.48*10^5^ |
| Total heat load | kCal /24 h | 1.5*10^6^ | 6.99*10^5^ |
| Total heat load | kCal /16h | 9.98*105 | 4.66*10^5^ |
| **Total heat load** | **kCal/h** | **6.2*10^4^** | **2.91*10^4^** |
| Service load (10% of the total heat load) | kCal /24 h | 1.5*10^5^ | 6.99*10^4^ |
| Service load (10% of the total heat load) | kCal /16 h | 1*10^5^ | 4.66*10^4^ |
| Service load (10% of the total heat load) | kCal / h | 6.2*10^3^ | 2.91*10^3^ |
| **Total heat load+service load** | **kCal/24 h** | **1.6*10^6^** | **7.69*10^5^** |
| **Total heat load+service load** | **kCal/16 h** | **1.1*10^6^** | **5.13*10^5^** |
| **Total heat load+service load** | **kCal/h** | **6.9*10^4^** | **3.2*10^4^** |
| Assumed operation of refrigeration | hours/day | 16 | 16 |
| Number of days for refrigeration | days | 14 | 7 |
| Refrigeration Capacity required | ton -refrigeration (tons-ref) | 22.68 | 10.59 |
| 1-ton refrigeration | kW per 1-ton ref | 3.52 | 3.52 |
| Total Refrigeration capacity | kWh/year | 1.79*10^4^ | 4.18*10^3^ |
| Refrigeration capacity per kg product | kWh per kg product | 1.79*10^-1^ | 4.18E*10-^2^ |
|  |  |  |  |
| **Assumptions:**  * Data for fresh tomatoes are shown in the case of storing fresh market tomatoes.  Heat of respiration  <https://www.ams.usda.gov/sites/default/files/media/TransportPerishableFoodsbyTruck%5B1%5D.pdf>  Specific heat <http://www.mpd-inc.com/bulk-density/>  Optimum cooling temperature, approximate storage life (in days. For potatoes, shown above: <https://extension.umaine.edu/publications/4135e/> | | | |

**Appendix 7**. Energy inputs at Retail (supermarkets).

Appendix 7.a. Calculation of energy inputs at Retail (supermarkets) (EPA, 2015; SEDAC, 2013)

| Inputs | Units | Amount |
| --- | --- | --- |
| Electricity | kWh/sq. ft | 50 |
| Natural gas | kWh/sq. ft | 1517 |

Appendix 7.b. Distribution of energy inputs at Retail (supermarkets) (SEDAC, 2013)

| Used for | Electricity | Natural gas |
| --- | --- | --- |
| Refrigeration | 57% | - |
| Lighting | 23% | - |
| Ventilation | 5% | - |
| Cooling | 5% | - |
| Heating | 2% | 69% |
| Computers | 1% | - |
| **Refrigerated** | **93%** | - |
| **Unrefrigerated** | **36%** | **-** |

Appendix 7.c. Area and numbers of supermarkets consider for calculating energy inputs (EPA, 2015)

| **Retail descriptions** | Supermarket |
| --- | --- |
| Total area (sq. ft) | 50009 |
| Numbers | 15639 |
| **Consumer-facing area**^a^ |  |
| Potato-chips (as, potato packaged) | 0.1% |
| Potato-dehydrated (as, potato packaged) | 0.1% |
| Potato-frozen fries | 0.3% |
| Tomato paste (as pasta packed) | 0.1% |
| **Assumptions:**  ^a^ Willard Bishop Total Store SuperStudyTM, May 2016 (https://superstudy.willardbishop.com) | |

Appendix 7.d. Average annual sales of the selected products (data represent the domestic availability sales for the years 2010-2018) (Parr and Daugherty, 2018). For tomato pasta, 80% of the total sales of the tomato processing was accounted.

| Used for | Unit | Potato-  chips | Potato-  Frozen fries | Potato-dehydrated | Tomato pasta |
| --- | --- | --- | --- | --- | --- |
| Annual average domestic availability | Million tons | 2.55 | 7.09 | 1.77 | 7.42 |
| Domestic availability per store per year | tons | 163 | 454 | 113 | 474 |
| **Assumptions:**  Total number of supermarkets = 15639. Total domestic availability was divided by number of supermarkets for accounting per store products’ volume. | | | | | |

Appendix 7.e. Average annual sales of the fresh potato and tomato that can be used for fresh market evaluation (data represent the domestic availability sales for the years 2010-2018) (Parr and Daugherty, 2018).

| Used for | Unit | Potato-  fresh | Tomato-  Fresh* |  |
| --- | --- | --- | --- | --- |
| Annual average domestic availability | Million tons | 4.92 | 2.97 |  |
| Domestic availability per store per year | tons | 315 | 190 |  |
| **Assumptions:**  * Fresh tomatoes data are shown to assist in modeling fresh market F&V products.  Total number of supermarkets = 15639. Total domestic availability was divided by number of supermarkets for accounting per store products’ volume. | | | | |

**Appendix 8.** Assumptions made for the transportation

| Distances | Units | Values |
| --- | --- | --- |
| Farm to farm store | km | 5 |
| Farm store to Warehouse (Processor) ^a^ | km | 267 |
| Processor to Retail^b^ | km | 1200 |
| Retail/Distributor to consumer^a^ | km | 10 |
| **Assumption:**  ^a^ Farm to Processor transportation distance, assumed after considering the average miles driven reported for potato and tomato, in the studies, including Refs. (Bureau of Transportation Statistics, 2017; Durham et al., 1995). See section 2.2.1, in the LCA Protocol.  ^b^ Average distance calculated based on the locations of the potato and tomato processing plants in the U.S., and taking a mid-point in a US map. The average distance assumed was 1200 km. | | |

**Appendix-9.** Processing of the potato to produce potato-chips and potato-frozen fries. Method for calculating the energy input as per the mass flow rates inside the fryer based on (Wu et al., 2010).

|  | Units | Amount | Remarks //methods |
| --- | --- | --- | --- |
| Water content ratio |  | 0.8 |  |
| Mass flows per hour |  |  |  |
| Mass of oil input (ṁ_1_) | kg | 62.84 |  |
| Mass of oil return (ṁ_2_) | kg | 60 |  |
| Fine products removal (ṁ_3_) | kg | 0.20 |  |
| Mass of air (ṁ_4_) | kg | 2.02 |  |
| Mass of frying vapors (ṁ_5_) | kg | 5.56 |  |
| Raw potato entering into the fryer (ṁ_6_) | kg | 0.99 |  |
| Mass of the fried potato (ṁ_7_) | kg | 0.95 | Appendix 10 |
| Surface water of the raw material (ṁ_sw_) | kg | 0.10 |  |
| Ambient temperature (T_amb_) | ^o^C | 24.85 |  |
| Temperature of the external surface of the fryer (T_fo_) | ^o^C | 172.85 |  |
| Air temperature (T_4_ = T_amb_) | ^o^C | 24.85 |  |
| Temperature of frying vapour (T_5_) | ^o^C | 102.75 |  |
| Temperature of the raw material (T_6_) | ^o^C | 59.85 |  |
| Temperature of the final fried product (T_7_) | ^o^C | 149.85 |  |
| Temperature of flowing water (T_b_) | ^o^C | 100 |  |
| Ratio of potato solid to mass flow rate 6 (x_s,6_) |  | 0.3 |  |
| Ratio of oil to mass flow rate 7 (x_o,7_) |  | 0.303 |  |
| Ratio of water content to mass flow  rate 7 (x_w,7_) |  | 0.016 |  |
| Ratio of potato solid to mass flow rate  7 (x_s,7_) |  | 0.34 | Calculated |
| Ratio of water content to mass flow  rate 6 (x_w,6_) |  | 0.8 | Calculated |
| Ratio of air to mass flow rate 5 (x_a,5_) |  | 0.36 | Calculated |
| Ratio of oil vapor to the mass flow rate 5 (x_v,5_) |  |  | Calculated |
| Latent heat of  water evaporation (h_w_) | kCal/kg | 2256.7 |  |
| Specific heat of air (c_pa_) | kJ/kg ^o^C | 1.01 |  |
| Specific heat of the potato solid (c_ps_) | kJ/kg ^o^C | 1.30 |  |
| Specific heat of water (c_pw_) | kJ/kg ^o^C | 4.18 |  |
| Latent heat of oil (h_0_) | kJ/kg ^o^C | 300 |  |
| Surface area of the casing (A) | m^2^ | 45.00 |  |
| Overall heat transfer coefficient of the casing of the fryer (U) | kW/m^2^ ^o^C | 0.014 |  |
| Heat energy input per hour |  | *ṁ* | Energy input for the mass flow |
| Energy needed to heat raw potato in fryer (E_1_) |  | 35 | E_1_ = c_ps_* *ṁ_6_** x_s,6_ * (T_7_- T_6_) |
| Energy needed to vaporize water (E_2_) |  | 2949 | E_2_ - [c_pw_ (T_b_-T_6_) + h_w_] * (*ṁ*_6_*x_w,6_ + *ṁ*_sw_ - *ṁ*_7_*x_w,7_) |
| Energy needed to heat oil (E_3_) |  | 706 | E_3_ = h_o_ (*ṁ*_1_ - *ṁ*_2_ - *ṁ*_3_- *ṁ*_7_*x_o,7_) |
| Energy needed to heat air (E_4_) |  | 157 | E_4_ =C_pa_* *ṁ*_4_*(T_5_-T_4_) |
| Energy transmitted through external surface to the fryer (E_5_) |  | 9.3 | UA (T_fo_ -T_amb_) |
| Total energy input per hour | kJ | 3854 | (3026 kJ for dehydrated, excluding heating oil and air heating) |

**Appendix 10.** Mass flow in the processors to produce potato-chips and potato-frozen fries. Basic parameters based on: Mass flow (Fooddelphi, 2018; Wu et al., 2010), energy inputs (Fooddelphi, 2018; Wu et al., 2010).

|  | Mass flow | Units | Potato-  Chips | Potato-  frozen fries | Potato-dehydrated |
| --- | --- | --- | --- | --- | --- |
|  | Raw potato | kg | 1.9 | 2.12 | 1.94 |
|  | **Subdivisions to respective line** |  |  |  |  |
| A | Destoning and washing |  |  |  |  |
|  | Destoned potato | kg | 1.89 | 2.11 | 1.93 |
|  | *Water* | kg | 2.67 | 2.98 | 2.72 |
| B | Steam for peeling |  |  |  |  |
|  | *Mass handled* | kg | 1.89 | 2.11 | 1.93 |
|  | *Steam, hourly* | kg | 0.76 | 0.84 | 0.77 |
|  | *Fuel used (64 kg fuel)* | kCal | 6.90 | 7.69 | 7.03 |
|  | *Power required for conveying peeled potato to slicing unit* | kWh | 1.39*10^-3^ | 1.55*10^-3^ | 1.42*10^-3^ |
|  | Output |  |  |  |  |
|  | *Peeled potato* | kg | 1.44 | 1.60 | 1.47 |
|  | *waste, peel* | kg | 0.45 | 0.50 | 0.46 |
| C | Power for slicing |  |  |  |  |
|  | *Mass of potato taken* | kg | 1.44 | 1.60 | 1.47 |
|  | *Power* | kWh | 9.96 | 11.09 | 10.14 |
|  | *Power required for conveying sliced potato to washing* | kWh | 7.1*10^-4^ | 7.26*10^-4^ | 7.23*10^-4^ |
|  | Output |  |  |  |  |
|  | *Sliced potato* | kg | 1.34 | 1.37 | 1.36 |
|  | *Waste, from slicing* | kg | 0.10 | 0.23 | 0.10 |
| D | Slice washing |  |  |  |  |
|  | *Mas handled* | kg | 1.34 | 1.37 | 1.36 |
|  | *Water* | kg | 0.80 | 0.69 | 0.82 |
|  | Output |  |  |  |  |
|  | *Potato slices* | kg | 1.31 | 1.34 | 1.33 |
|  | *Wastewater* | kg | 0.80 | 0.69 | 0.82 |
|  | *Starch* | kg | 0.03 | 0.03 | 0.03 |
| E | Blanching |  |  |  |  |
|  | *Mass handled* | kg | - | 1.34 | 1.33 |
|  | *Power required for conveying from slicing unit to blanching level* | kWh | - | 3.61*10^-4^ | 3.6*10^-4^ |
|  | *Water* | kg | - | 2.1 | 2 |
|  | Output |  |  |  |  |
|  | *Blanched potato* | kg | - | 1.12 | 1.11 |
|  | *Waste water* | kg | - | 2.00 | 1.99 |
|  | *Starch* | kg | - | 0.23 | 0.22 |
| F | Dewatering |  |  |  |  |
|  | *Mass handled* | kg | 1.30 | 1.30 | 1.30 |
|  | *Water removed* | kg | 4.93E-03 | 6.62*10^-3^ | 6.59*10^-3^ |
|  | *Power required for conveyor* | kW-h | 4.44E-05 | 3.04*10^-5^ | 3.03*10^-5^ |
| G | Frying |  |  |  |  |
|  | *mass handled* | kg | 1.30 | 1.30 | - |
|  | *palm oil* | kg | 0.43 | 0.21 | - |
|  | *Heat supplied* | kW | 45.54 | 45.54 | - |
|  | *power required to pump oil* | kW-h | 1.35*10^-3^ | 6.54*10^-4^ | - |
|  | *Output* |  |  |  |  |
|  | *Fried potato, before salting* | kg | 1.30 | 1.30 | - |
|  | *Fried potato, with 33% oil , and 16% oil* | kg | 1.67 | 1.50 | - |
|  | *Oil waste* | kg | 0.06 | 0.01 | - |
| H | Salting |  |  |  |  |
|  | *Mass handling capacity* | kg | 1.67 | - | - |
|  | *Power required to convey fried potato to salting* | kW-h | 5.32*10^-5^ | - | - |
|  | *Salt* | kg | 0.04 | - |  |
|  | *Output, fried potato, with 2% salt* | kg | 1.71 | - | - |
|  | *Power required to convey fried potato to pre-cooling unit* |  | - | 1.21*10^-3^ | - |
| I | Pre-cooling |  |  |  |  |
|  | Mass handled, without oil |  | - | - | - |
|  | *mass handled* | kg | - | 1.5 | - |
|  | *Power required for conveyor type pre-cooler* | kWh | - | 2.81*10^-4^ | - |
|  | *Power required for conveying from precooler to freezing unit* | kWh | - | 1.59*10^-3^ | - |
|  | *Pre-cooling and freezing load* | kWh | - | 0.27 | - |
|  | *Packaging* |  |  |  |  |
|  | *Mass handling capacity* | kg | 1.67 | 1.5 | 1.3 |
|  | *Power required for conveying after packaging* | kW-h | 5.79*10^-4^ | 1.19*10^-3^ | 4.51*10^-4^ |
|  | **Total power required** |  |  |  |  |
|  | *Total power required for all conveyors* |  | 3.29*10^-3^ | 6.94*10^-3^ | 2.98*10^-3^ |
|  | Storing, frozen fries in cold storage, heat load |  |  |  |  |
|  | Total refrigeration capacity-pre-cooling | kW | - | 2.72*10^-1^ | - |
|  | Total refrigeration capacity-cold store | kW | - | 1.94*10^-3^ | - |
|  | Electricity for operating pre-cooler and cold storage | kWh | - | 7.08*10^-1^ | - |
|  | Power required for drying | kWh | - | - | 1.55*10^-1^ |
|  | **Assumptions:**  Drying of potato takes place after dewatering of the sliced potatoes.  Assumed to be carried out in a multistage rotary air dryer: Stage 1 (section 1, air dried from ambient temperature to 102 ^o^C, section-2: ambient temperature to 88 ^o^C), Stage 2: ambient temperature to 68.5 ^o^C.  Heat energy required for drying dryer surface (air drying) = m_air_ * cpa * (t_amb_-T-stages_i,j,k_) (see texts). Mass of air required for 1 kg potato-product and specific heat capacity shown in Appendix 10. | | | | |

**Appendix 11.** Calculation of the freezing load for the potato-frozen fries, based on (Fooddelphi, 2018). For calculation purpose, initially 1000 kg was selected as the mass to be handled per hour.

|  | Amount | Units | Parameters | values | Units |
| --- | --- | --- | --- | --- | --- |
| Mass of frozen potato fries (pfr) | 1000 | kg |  |  |  |
|  |  |  |  |  |  |
| Power reqd. to cool product (after frying) | Q1 | **Q1 = m_ps_*c_p-pfr_* (t_fr_-t_r_)** | | | |
| Q1 | 86.89 | kW | cp_pfr_ = | 3.33 | kf/kg ^o^C |
|  |  |  | Temperature of fries (t_fr_) = | 85 | ^o^C |
|  |  |  | Product temperature drop down (t_r_) = | 2 | ^o^C |
| Power required to cool air from 25 to 0 ^o^c | Q2 | **Q2 = m_ps_*c_pa_* (t_amb_-t_c)_** | | | |
| Q2 | 31.98 | kW | Ambient temperature (T_amb_) = | 25 |  |
|  |  |  | Temperature required before frezing (T_c_) = | 0 |  |
|  |  |  | Cpa = | 1.005 |  |
|  |  |  | mass of air = | 1273 | kg |
|  |  |  |  |  |  |
| Cooling to freezing point, at -1.7 ^o^c | Q3 | **Q3 = m_ps_*c_p-fr_* (t_pc_-t_frz1_)** | | | |
| Q3 | 4.09 | kW | T_precool_ (t_pc_) | 2 |  |
|  |  |  | Tfreez (t_frz1_) = | -1.7 |  |
|  |  |  | cp_pfr_ (specific heat of potato frozen fries) = | 3.52 | kJ/kg |
|  |  |  | mass of product = | 1000 | kg |
|  |  |  |  |  |  |
| Freezing, at freezing stage 2 (tfrz2) -1.7 ^o^c | Q4 | **Q4 = m_ps_ * ƛ** | | | |
| Q4 | 75.73 | kW | ƛ = | 240.89 | kJ/kg |
|  |  |  | m = | 1000 | kg |
|  |  |  |  |  |  |
| Cooling from freezing point to final temperature | Q5 | **Q5 = m_ps_*c_p-fr_* n*ρ *s* (t_fin_-t_frz2_)** | | | |
| Q5 | 5.75 | kW | t_fin_ = | -18 |  |
|  |  |  | tf_rz2_ = | -1.7 |  |
|  |  |  | cp_-pfr =_ | 1.76 |  |
|  |  |  | ρ = | 0.91 |  |
|  |  |  | n = | 0.8 |  |
|  |  |  | mps = | 1000 |  |
|  |  |  |  |  |  |
| **Cold storage design** |  |  |  |  |  |
| Surface area (A) | 50 | m^2^ |  |  |  |
| U-materials |  | **U = 1/ [(1/ho) + ( 1/hi) + (Δxy/ky) + (Δxp/kp)]** | | | |
| **Building materials** |  |  | | | |
| **Walls** |  |  |  |  |  |
| U-walls | 0.34 | W/m^2^.K | T_outside_ = | 10.4 | ^o^C |
| Q-walls | 579 | W | T_outside-walls_ = | 16.4 | 6 ^o^C, for sun exposure |
|  |  |  | T_inside_ = | -18 |  |
| **Roof** |  |  | h_inside_ = | 9.37 | W/m^2^.K |
| U-roof | 0.35 |  | h_outside_ = | 22.7 | W/m^2^.K |
| Q-roof | 755 | W | k-ytong = | 0.13 | W/m. K |
|  |  |  | k-polyuretan = | 0.12 | W/m. K |
|  |  |  | ∆x-ytong = | 0.15 | M |
|  |  |  | ∆x-polyuretan = | 0.2 | M |
|  |  |  | T_outside-roof_ = | 25.4 | max temps |
|  |  |  | h_outside-roof_ = | 60.07 | W/m^2^.K |
|  |  |  | h_inside-roof_ = | 27.58 | W/m^2^.K |
| Q air change load |  |  |  |  |  |
| Q-air-change | 0.98 | W | mass of fries = | 1000 | kg/hr |
|  |  |  | Specific heat of frozen fries  (c_p-fr_) = | 3.52 | kJ/kg |
|  |  |  | T1 = | -17 |  |
|  |  |  | T2 = | -18 |  |
| Subtotal (Q-sub- total) | 1335 | W |  |  |  |
| Service load (Q-service) | 133.5 | W | 10% of the total heat load |  |  |
| Total heat load (Q-total) | 1.47 | kW |  |  |  |
| Refrigeration Capacity |  |  |  |  |  |
| Refrigeration Capacity required | 0.42 | Ton -Ref | Assumed operation of refrigeration = | 16 | hrs/day |
|  |  |  | Number of days for refrigeration = | 14 | Days |
|  |  |  | 1 ton refrigeration = | 3.52 | kW per 1 ton ref |
| **Cold storage** |  |  |  |  |  |
| Electricity consumption for total capacity | 329 | kWh |  |  |  |
| Electricity consumption per kg product | 0.33 | kWh |  |  |  |
| **Precooler** |  |  |  |  |  |
| Electricity consumption for pre-coolers | 205 | kWh |  |  |  |
| Refrigeration Capacity required | 58.35 | Ton-ref |  |  |  |
| Electricity consumption per kg product | 0.205 | kWh |  |  |  |

**Appendix 12. Biowaste handling scenarios**

|  | Biowaste management | Remarks |
| --- | --- | --- |
| Basic/baseline scenario | Biowaste:   - Farm to retail waste assumed undergoing composting - Unwanted potatoes, peeled potatoes and residues from processing plant and products loss at retail were considered for alternative livestock feed - Consumer waste followed: *composting (7%), combustion with energy recovery (18%) and landfill (75%)* (EPA, 2018) - Recovered starch (for potatoes): as an alternative supply to the market | Corn feed is assumed to be substituted (Schroeder, 2012; Wadhwa et al., 2015). Details on the related parameters considered for the estimates shown in Appendix – 12-13  Corn-starch was selected as substitutable starch (Stearns et al., 1994) |
| Bio-waste management scenario-1 | - 100% biowaste generated across the supply chain undergoing composting - Recovered starch- same as basic scenario | Consequences of substitution of the NPK-synthetic, at the farm level included. Details on the related parameters considered for the estimates shown in Appendix – 13-14  All N-related emissions (direct and indirect) due to addition of compost and the substitution of synthetic fertilizers were accounted |
| Bio-waste management scenario-2 | - Farm to retail waste (compost); and rest of the biowaste generated across the supply chain undergoing anaerobic conversion and combustion of biogas in a CHP plant, producing heat and electricity - Recovered starch- same as basic scenario | Substitution of marginal heat (natural gas) and electricity mix of the U.S included. Details on the related parameters considered for the estimates shown in Appendix – 13-14 |
| Bio-waste management scenario-3 | - 100% of biowaste undergoing treatment process, as suggested in (EPA, 2018) and EPA (facts and figures. It covered: composting (7%), combustion with energy recovery (18%) and landfill (75%) - Recovered starch- same as basic scenario | Waste treatment model adopted from Ecoinvent v3.6 |

**Appendix 13.** Parameters considered for calculating the alternative values of the waste in the selected scenarios.

| Parameters | Values | Remarks |
| --- | --- | --- |
| **Waste to fertilizer** ^a^ |  | See footnotes^a^ |
| DM, at harvest | 6%, 20% | Tomato and potato |
| N, % | 0.7 |  |
| P, % | 0.4 |  |
| Nutrient uptake, eff, % | 0.15 ^a^ |  |
| **Waste to feed** ^b^ |  | See footnotes^b^ |
| Carbohydrate%/starch | 50%, 70% | Tomato and potato |
| Protein value (kg, crude protein) | 0.221, 0.095 | Tomato and potato |
| DM (average of waste streams) at processor | 40% (potato) and 21% (tomato sauce) |  |
| NDF | 26.5%, 63% | Tomato and potato |
| Metabolize energy (ME) (MJ/kg DM) | 0.005, 0.0067 | Tomato and potato |
| **Waste to energy** ^c^ |  | See footnotes^c^ |
| VS per kg DM | 0.546 |  |
| Biogas yield (m^3^ biogas per kg VS) | 0.426 |  |
| Methane content in the biogas (%) | 70% |  |
| MJ per m^3^ CH_4_ | 35.8 |  |
| MJ per m3 natural gas | 36.6 |  |
| **Assumptions:**  ^a^ **Waste to fertilizer:**  NPK values and mass reduction of the bio-waste during composting 50% (Wernet et al., 2016) and (Mukta et al., 2015).  Fertilizer equivalent for compost and synthetic fertilizer, for N = 15% (Horrocks et al., 2016). N and P concentrations (Li et al., 2017; Mukta et al., 2015; Sarkar et al., 2016).  ^b^ **Waste to feed:**  Feed values based on**:** (Wadhwa and Bakshi, 2013)**.** DM content of potato products and tomato sauce assumed at 40% and 21% respectively.  ^c^ **Waste to energy**  Volatile Substance (VS) averaged from values reported in Refs. (Bouallagui et al., 2003; Dhanalakshmi Sridevi et al., 2015; Gunaseelan, 1997; Kübler et al., 2000; Mata-Alvarez et al., 1992; Paritosh et al., 2017; Patil and Deshmukh, 2015)  Methane content: (Bouallagui et al., 2003).  Digestate N-values = 2.4 g/kg fresh digestate.  <http://americanbiogascouncil.org/adCoProductsResources/IEA_digestate_Use_manual.pdf> | | |

**Appendix 14.** Parameters for biogas conversion in CHP plants

|  | Units | Values |
| --- | --- | --- |
| **Power plant efficiency^a^** |  |  |
| heat, eff | % | 55% |
| elect, eff | % | 35% |
| Overall eff. | % | 80% |
| **Combustion input^b^** | |  |
| Heat | MJ per MJ CH_4_ | 1.84*10^-2^ |
| Electricity | MJ per MJ CH_4_ | 2.8*10^-3^ |
| **Emission CH_4_^b^** |  |  |
| CH_4_ from plant | % | 1.8% |
| CH_4_ during combustion | kg per m^3^ | 7.4*10^-3^ |
| N_2_O during combustion | kg per m^3^ | 1.2*10^-5^ |
| **Assumptions:**  ^a^ Heat and electricity efficiency: (NREL, 2010)  ^b^ Inputs for combustion and emissions: (Parajuli et al., 2018) and (Pugesgaard et al., 2014). Conversion factor: m^3^ CH_4_ to kg = 0.67 | | |

**Appendix-15**. Avoided emissions due to use of digestate as N fertilizer in the waste-to-energy scenario

|  |  | Potato products | | | Tomato product |
| --- | --- | --- | --- | --- | --- |
|  |  | Potato-chips | Potato-frozen  Fries | Potato  dehydrated | Tomato-sauce |
| Reference Unit | kg | 1 | 1 | 1 | 1 |
| Biowaste^a^ | kg | 1.05 | 1.14 | 1.05 | 9.46**10^-1^* |
| Digestate (N-values) | kg | 2.71E-04 | 3.16E-04 | 2.71E-04 | 1.04**10^-4^* |
| Avoided emissions |  |  |  |  |  |
| Total N_2_O-N^b^ | kg | -6.23*10^-6^ | -7.27*10^-6^ | -6.23*10^-6^ | -2.39**10^-6^* |
| (a) Calculation steps for N_2_O (direct) | kg | -3.39*10^-6^ | -3.95*10^-6^ | -3.39*10^-6^ | -1.3**10^-6^* |
| *N2O-N (N-synthetic application)* | *kg* | -3.39*10^-6^ | -3.95*10^-6^ | -3.39*10^-6^ | -1.3**10^-6^* |
| (b) Calculation steps for N_2_O (indirect)-N emissions | kg | -2.84*10^-6^ | -3.32*10^-6^ | -2.84*10^-6^ | -1.09*10^-6^ |
| *NH_3_-N (from N-synth application)* | *kg* | -6.77*10^-6^ | -7.91*10^-6^ | -6.77*10^-6^ | -2.60*10^-6^ |
| *NOx-N (from N-synth application)* | *kg* | -2.37*10^-6^ | -2.77*10^-6^ | -2.37*10^-6^ | -9.09*10^-7^ |
| NO_3_-N (potential leaching) | *kg* | -3.67*10^-4^ | -4.29*10^-4^ | -3.67*10^-4^ | -1.41**10^-4^* |
| Added emissions (added digestate) |  |  |  |  |  |
| Total N_2_O-N ^b^ | kg | 4.64*10^-6^ | 5.42*10^-6^ | 4.64*10^-6^ | 1.78**10^-6^* |
| (a) Calculation steps for N_2_O (direct) | kg | 2.71*10^-6^ | 3.16*10^-6^ | 2.71*10^-6^ | 1.04**10^-6^* |
| *N_2_O-N (N-digestate)* | *kg* | 2.71*10^-6^ | 3.16*10^-6^ | 2.71*10^-6^ | 1.04**10^-6^* |
| (b) Calculation steps for N_2_O (indirect)-N emissions | kg | 1.93*10^-6^ | 2.26*10^-6^ | 1.93*10^-6^ | 7.42*10^-7^ |
| *NH_3_-N (from N-digestate application)* | *kg* | 5.42*10^-6^ | 6.33*10^-6^ | 5.42*10^-6^ | 2.08*10^-6^ |
| *NOx-N (from N-digestate application)* | *kg* | 1.90*10^-6^ | 2.21*10^-6^ | 1.90*10^-6^ | 7.27*10^-7^ |
| NO3-N (potential leaching) | kg | 2.48*10^-4^ | 2.90*10^-4^ | 2.48*10^-4^ | 9.51*10^-5^ |
| Net emissions |  |  |  |  |  |
| N_2_O | kg | -2.49*10^-6^ | -2.91*10^-6^ | -2.49*10^-6^ | -6.09*10^-7^ |
| NH_3_ | kg | -1.64*10^-6^ | -1.91*10^-6^ | -1.64*10^-6^ | -5.19*10^-7^ |
| NOx | kg | -1.56*10^-6^ | -1.82*10^-6^ | -1.56*10^-6^ | -1.82*10^-7^ |
| NO_3_ | kg | -5.27*10^-4^ | -6.15*10^-4^ | -5.27*10^-4^ | -4.56*10^-5^ |
| **Assumptions:**  ^a^ Biowaste for waste to energy conversion = the total post-harvest biowaste generated across the supply chain. Fertilizer value of the digestate (see Appendix 13).  ^b^ Calculation steps for emissions followed the IPCC guideline (IPCC, 2006) (see Table 3 in the main document). Net impact was estimated considering added emissions from compost and negative emissions due to the substitution of N-synthetic fertilizer.  ^c^ P-emissions estimation based on LCI guideline (Nemecek et al., 2015). Net emissions are calculated to be zero (indicated as (“±”) above, since P use efficiency of added compost and substituted synthetic P fertilizer was set to 100% (resulting to net emissions as zero) (Nguyen et al., 2013). | | | | | |

**Appendix-16**. Waste management scenarios considered in the LCA model

| Waste^a^ | Waste treatment scenario | | | |
| --- | --- | --- | --- | --- |
|  | Landfilling | Incineration | Recycling | Composting |
| Plastic waste | 75.4% | 15.5% | 9.1% | - |
| Paper^b^ | 72%^b^ | 6.5% | 21%^b^ | - |
| Corrugated box^b^ | 72%^b^ | 6.5% | 21%^b^ | - |
| Glass | 60.8% | 12.8% | 26.4% | - |
| Metal caps/Aluminum | 67.6% | 13.9% | 18.5% | - |
| Food waste/Biowaste^b^  (in scenario-3)^±^ | 76.1% | 18.6% | - | 5.3% |
| Assumptions:  ^a^ Data based on: (EPA, 2018) and EPA (facts and figures)  <https://www.epa.gov/facts-and-figures-about-materials-waste-and-recycling/national-overview-facts-and-figures-materials>.  ^b^ Available paper and corrugated box for recycling = 93% of the generated waste. Of the available waste, 48% are recyclable. Considering the 48% of the generated waste assumed for recycling, recyclable corrugated box hence would be 22%. Sorted waste further assumed for landfilling (<https://www.corrugated.org/wp-content/uploads/PDFs/White_Papers/CPA_Recycling_White_Paper_August2016.pdf>)  ^c^ Biowaste (in the baseline scenario): composting (for consumer waste) and rest of the biowaste (as animal feed). ± In the alternative scenario (scenario-3), the composition of waste management scenarios is based on (EPA, 2018). | | | | |

# References-Supporting information

ASTM Standards, 2018. Paper standards and packaging standards. https://www.astm.org/Standards/paper-and-packaging-standards.html (accessed Oct 22, 2018).

Bouallagui, H., Cheikh, R. Ben, Marouani, L., Hamdi, M., 2003. Mesophilic biogas production from fruit and vegetable waste in a tubular digester. Bioresour. Technol. 86, 85–89. https://doi.org/10.1016/S0960-8524(02)00097-4

Bureau of Transportation Statistics, 2017. 2017 CFS Preliminary Data [WWW Document]. URL https://www.bts.gov/surveys/commodity-flow-survey/2017-cfs-preliminary-data (accessed Nov 5, 2019)

Buzby, J.C., Hyman, J., 2012. Total and per capita value of food loss in the United States. Food Policy 37, 561–570. https://doi.org/10.1016/j.foodpol.2012.06.002

Buzby, J.C., Hyman, J., 2012. Total and per capita value of food loss in the United States. Food Policy 37, 561–570. https://doi.org/10.1016/j.foodpol.2012.06.002

Buzby, J.C., Wells, H.F., Axtman, B., Mickey, J., 2009. Supermarket loss estimates for fresh fruit, vegetables, meat, poultry, and seafood and their use in the ERS loss-adjusted food availability data. Econ. Inf. Bull.-USDA Econ. Res. Serv. https://www.ers.usda.gov/publications/pub-details/?pubid=44309 (accessed Feb 25, 2018)

Carvalho, M., Grilo, M.M. de S., Abrahao, R., 2018. Comparison of greenhouse gas emissions relative to two frying processes for homemade potato chips. Environ. Prog. Sustain. Energy 37, 481–487. <https://doi.org/10.1002/ep.12661>

Dhanalakshmi Sridevi, V., Rema, T., Srinivasan, S. V, 2015. Studies on biogas production from vegetable market wastes in a two-phase anaerobic reactor. Clean Technol. Environ. Policy 17, 1689–1697. https://doi.org/10.1007/s10098-014-0883-8

Durham, C., Sexton, R., Song, J., 1995. Optimizing tomato distribution to processors lifts profits little. Calif. Agric. 49, 21–26. https://doi.org/10.3733/ca.v049n05p21.

Elik, A., Yanik, D.K., Istanbullu, Y., Guzelsoy, N.A., Yavuz, A., Gogus, F., 2019. Strategies to reduce post-harvest losses for fruits and vegetables. International Journal of Scientific and Technological Research, 1-29. DOI: 10.7176/JSTR/5-3-04 Vol.5, No.3, 2019

EPA, 2018. Advancing sustainable materials management: 2014 fact sheet. U.S. Environmental Protection Agency (EPA), pp 1-22. https://www.epa.gov/sites/production/files/2018-07/documents/2015_smm_msw_factsheet_07242018_fnl_508_002.pdf (Accessed Jan 25, 2019)

EPA, 2015. Energy Use in Supermarkets. Energy Star Portfolio Manager. https://www.energystar.gov/sites/default/files/tools/DataTrends_Supermarket_20150129.pdf (accessed May 3, 2019)

Eranki, P.L., El-Shikha, D., Hunsaker, D.J., Bronson, K.F., Landis, A.E., 2017. A comparative life cycle assessment of flood and drip irrigation for guayule rubber production using experimental field data. Ind. Crops Prod. 99, 97–108. https://doi.org/10.1016/j.indcrop.2017.01.020

Fooddelphi, 2018. Filtration and Cold Storage Calculations. URL https://www.foodelphi.com/filtration-and-cold-storage-calculations/ (accessed Nov 10, 2018)

Gunaseelan, V.N., 1997. Anaerobic digestion of biomass for methane production: A review. Biomass Bioenergy 13, 83–114. https://doi.org/10.1016/S0961-9534(97)00020-2

Harvey, J.M., 1978. Reduction of losses in fresh market fruits and vegetables. Annu. Rev. Phytopathol. 16, 321–341. https://vric.ucdavis.edu/pdf/fertilization/fertilization_EfficientNitrogenManagementforCoolSeasonvegetable2017.pdf (accessed Sep 15, 2018)

Holmer, R.J., Schnitzler, W.H., 1997. Drip irrigation for small-scale tomato production in the tropics. Kasetsart JNat Sci 32, 56–60.

Horrocks, A., Curtin, D., Tregurtha, C., Meenken, E., 2016. Municipal compost as a nutrient source for organic crop production in New Zealand. Agronomy 6, 35. <https://doi.org/10.3390/agronomy6020035>

IPCC, 2006. 2006 IPCC Guidelines for National Greenhouse Gas Inventories. Volume 4. Chapter 5, IIPCC. http://www.ghgprotocol.org/sites/default/files/ghgp/hfc-cfc_0.pdf (accessed Sep 15, 2018)

Kenneth, G., Wang, C.Y., Mikal, S., 2016. The commercial storage of fruits, vegetables, and florist and nursery stocks. USDA, ERS, Agriculture Handbook Number 66, pp 1-792. https://www.ars.usda.gov/ARSUserFiles/oc/np/CommercialStorage/CommercialStorage.pdf (accessed Sep 10, 2017)

Kraemer, R., Plouff, A., Venn, J., 2015. Design of a Small-Scale, Low-Cost Cold Storage System. BE 487: Biosystems Design Project, pp 1-94. http://www.canr.msu.edu/hrt/uploads/535/78622/SOF-Cold-Cellar-Final-Report-94pgs.pdf (accessed Oct 20,2018)

Kübler, H., Hoppenheidt, K., Hirsch, P., Kottmair, A., Nimmrichter, R., Nordsieck, H., Mücke, W., Swerev, M., 2000. Full scale co-digestion of organic waste. Water Sci. Technol. 41, 195–202.

Li, S., Li, J., Zhang, B., Li, D., Li, G., Li, Y., 2017. Effect of different organic fertilizers application on growth and environmental risk of nitrate under a vegetable field. Sci. Rep. 7, 17020. https://doi.org/10.1038/s41598-017-17219-y

Mata-Alvarez, J., Cecchi, F., Llabrés, P., Pavan, P., 1992. Anaerobic digestion of the Barcelona central food market organic wastes. Plant design and feasibility study. Bioresour. Technol. 42, 33–42. https://doi.org/10.1016/0960-8524(92)90085-C

Maughn, T., Allen, N., Drost, D., 2017. Drip Irrigation for Commercial Vegetable and Fruit Production.

Minor, T., Astill, G., Raszap, S., Thornsbury, S., Buzby, J.C., Hitaj, C., Kantor, L., Kuchler, F., Ellison, B., Mishra, A.K., 2020. Economic Drivers of Food Loss at the Farm and Pre-Retail Sectors: A Look at the Produce Supply Chain in the United States, pp 1-39. <https://www.ers.usda.gov/publications/pub-details/?pubid=95778> (accessed Mar 21, 2018)

Mitchell, J.P., Shrestha, A., Klonsky, K., Turini, T.A., Hembree, K.J., 2014. Overhead and drip irrigation system effects on tomato growth and yield in California’s Central Valley. HortTechnology 24, 637–644. <https://doi.org/10.21273/HORTTECH.24.6.637>

Mouron, P., Willersinn, C., Möbius, S., Lansche, J., 2016. Environmental profile of the Swiss supply chain for French fries: Effects of food loss reduction, loss treatments and process modifications. Sustainability 8, 1214. 10.1016/j.agsy.2012.07.004

Mukta, S., Rahman, M.M., Mortuza, M.G., 2015. Yield and Nutrient Content of Tomato as Influenced by the Application of Vermicompost and Chemical Fertilizers. J. Environ. Sci. Nat. Resour. 8, 115–122. <https://doi.org/10.3329/jesnr.v8i2.26877>

Munoz-Carpena, R., Bryan, H., Klassen, W., Dukes, M.D., 2003. Automatic soil moisturebased drip irrigation for improving tomato production, in: Proceedings of the Florida State Horticultural Society. pp. 80–85.

Nemecek, T., Bengoa, X., Rossi, V., Humbert, S., Lansche, J., Mouron, P., Riedener, E., 2015. World Food LCA Database: Methodological guidelines for the life cycle inventory of agricultural products. Quantis Agroscope Version 3, 1–80. <http://link.ira.agroscope.ch/en-US/publication/34029> (accessed Mar 21, 2018)

Nguyen, T.L.T., Hermansen, J.E., Mogensen, L., 2013. Environmental performance of crop residues as an energy source for electricity production: The case of wheat straw in Denmark. Appl. Energy 104, 633–641. https://doi.org/10.1016/j.apenergy.2012.11.057

Nilsson, K., Sund, V., Florén, B., 2011. The environmental impact of the consumption of sweets, crisps and soft drinks. Nordic Council of Ministers, pp1-57. http://www.diva-portal.org/smash/get/diva2:702819/FULLTEXT01.pdf (accessed Mar 22, 2019)

NREL, 2010. 1–10 kW Stationary Combined Heat and Power Systems Status and Technical Potential . Independent Review Published for the U.S. Department of Energy Hydrogen and Fuel Cells Program, pp 1-40. https://www.nrel.gov/docs/fy10osti/48265.pdf (accessed Mar 22,, 2018)

Parajuli, R., Dalgaard, T., Birkved, M., 2018. Can farmers mitigate environmental impacts through combined production of food, fuel and feed? A consequential life cycle assessment of integrated mixed crop-livestock system with a green biorefinery. Sci. Total Environ. 619–620, 127–143. https://doi.org/10.1016/j.scitotenv.2017.11.082

Paritosh, K., Kushwaha, S.K., Yadav, M., Pareek, N., Chawade, A., Vivekanand, V., 2017. Food waste to energy: an overview of sustainable approaches for food waste management and nutrient recycling. BioMed Res. Int. 2017. <https://doi.org/10.1155/2017/2370927>

Parr, B., Daugherty, K., 2018. Vegetables and Pulses Outlook, April 2018. USDA, ERS. https://www.ers.usda.gov/data-products/vegetables-and-pulses-data/documentation.aspx (accessed Apr 15, 2019)

Patil, V.S., Deshmukh, H. V, 2015. Anaerobic digestion of Vegetable waste for Biogas generation: A Review. Int. Res. J. Environ. Sci. 4, 80–83. www.isca.in, [www.isca.me](http://www.isca.me)

Pugesgaard, S., Olesen, J.E., Jørgensen, U., Dalgaard, T., 2014. Biogas in organic agriculture—effects on productivity, energy self-sufficiency and greenhouse gas emissions. Renew. Agric. Food Syst. 29, 28–41. <https://doi.org/10.1017/S1742170512000440>

Sarkar, S., Pal, S., Chanda, S., 2016. Optimization of a Vegetable Waste Composting Process with a Significant Thermophilic Phase. Procedia Environ. Sci. 35, 435–440. https://doi.org/10.1016/j.proenv.2016.07.026

Schroeder, K., 2012. Feeding Cull Potatoes to Dairy and Beef Cattle. University of Wisconsin Extension, Portage County 1–4. https://shaverlab.dysci.wisc.edu/wp-content/uploads/sites/204/2015/04/FeedingCullPotatoestoDairyandBeefCattle10-24-12.pdf (accessed Dec 21, 2018)

Schwankl, L., Grattan, S., Miyao, G., 1991. Subsurface drip irrigation of tomatoes: Drip system design, management promote seed emergence. Calif. Agric. 45, 21–23. https://doi.org/10.3733/ca.v045n06p21

SEDAC, 2013. SEDAC Energy Smart Tips. Supermarkets. The Smart Energy Design Assistance Center, https://smartenergy.illinois.edu/sites/smartenergy.illinois.edu/files/2017-05/EST_Supermarkets.pdf (accessed May 2, 2019)

Simonne, E., Hochmuth, R., Breman, J., Lamont, W., Treadwell, D., Gazula, A., 2008. Drip-irrigation systems for small conventional vegetable farms and organic vegetable farms. Univ. Fla. IFAS Ext., pp 1-24. https://edis.ifas.ufl.edu/pdffiles/hs/hs38800.pdf (accessed Feb 21, 2018)

Stearns, L.D., Petry, T.A., Krause, M.A., 1994. Potential food and nonfood utilization of potatoes and related byproducts in North Dakota. No. 1189-2016-94269. 1994, p 1-60. https://ageconsearch.umn.edu/bitstream/23364/1/aer322.pdf (accessed Dec 15, 2018)

Krishnakumar, T., 2002. Design of cold storage for fruits and vegetables. Tamil Nadu Agricultural University. 1–58. https://doi.org/13140/RG.2.2.14335.82082

USDA, 2019. Food Availability (Per Capita) Data System-Loss Adjusted Food Availability. [https://www.ers.usda.gov/data-products/food-availability-per-capita-data-system/ (Apr 15, 2018)

Voss, R.E., Baghott, K.G., Timm, H., 2001. Proper environment for potato storage. Vegetable Research and Information Center. The University of California. https://vric.ucdavis.edu/pdf/potatoes/potato_storage.pdf (accessed Oct 22, 2018)

Vzw, V., 2014. Overview of standard industrial packaging weights. Belgium. http://www.valipac.be/Belgium/members-info/pdf/OVERVIEW-OF-STANDARD-WEIGHTS.pdf (accessed Aug 30, 2018)

Wadhwa, M., Bakshi, M.P.S., 2013. Utilization of fruit and vegetable wastes as livestock feed and as substrates for generation of other value-added products. RAP Publication 2013/04. FAO Rome., Rap Publication, pp 1-67. http://www.fao.org/3/a-i3273e.pdf (accessed Jan 15, 2018)

Wadhwa, M., Bakshi, M.P.S., Makkar, H.P.S., 2015. Waste to worth: fruit wastes and by-products as animal feed. CAB Rev. 10, 1–26. http://www.cabi.org/cabreviews

Wernet, G., Bauer, C., Steubing, B., Reinhard, J., Moreno-Ruiz, E., Weidema, B., 2016. The ecoinvent database version 3 (part I): overview and methodology. Int. J. Life Cycle Assess. 21, 1218–1230. https://doi.org/10.1007/s11367-016-1087-8

Wu, H., Jouhara, H., Tassou, S.A., Karayiannis, T.G., 2010. Analysis of energy use in crisp frying processes. SEEP2010 Conference Proceedings, June 29th – July 2nd, Bari, ITALY. Politecnico di Bari-BB Press, pp. 1–8.
